# Orbital angular momentum and beyond in free-space optical communications

**DOI:** 10.1515/nanoph-2021-0527

**Published:** 2021-12-14

**Authors:** Jian Wang, Jun Liu, Shuhui Li, Yifan Zhao, Jing Du, Long Zhu

**Affiliations:** Wuhan National Laboratory for Optoelectronics and School of Optical and Electronic Information, Huazhong University of Science and Technology, Wuhan 430074, Hubei, China

**Keywords:** free-space optical communications, modulation, multicasting, multiplexing, orbital angular momentum, structured light

## Abstract

Orbital angular momentum (OAM), which describes tailoring the spatial physical dimension of light waves into a helical phase structure, has given rise to many applications in optical manipulation, microscopy, imaging, metrology, sensing, quantum science, and optical communications. Light beams carrying OAM feature two distinct characteristics, i.e., inherent orthogonality and unbounded states in principle, which are suitable for capacity scaling of optical communications. In this paper, we give an overview of OAM and beyond in free-space optical communications. The fundamentals of OAM, concept of optical communications using OAM, OAM modulation (OAM modulation based on spatial light modulator, high-speed OAM modulation, spatial array modulation), OAM multiplexing (spectrally efficient, high capacity, long distance), OAM multicasting (adaptive multicasting, *N*-dimensional multicasting), OAM communications in turbulence (adaptive optics, digital signal processing, auto-alignment system), structured light communications beyond OAM (Bessel beams, Airy beams, vector beams), diverse and robust communications using OAM and beyond (multiple scenes, turbulence-resilient communications, intelligent communications) are comprehensively reviewed. The prospects and challenges of optical communications using OAM and beyond are also discussed at the end. In the future, there will be more opportunities in exploiting extensive advanced applications from OAM beams to more general structured light.

## 1 Introduction

Structured light beams are a variety of special light fields that have tailored spatial structure with variant amplitude, phase and polarization distribution [1], [2], [3], such as helically-phased beams, Bessel beams, Airy beams, vector beams and spatiotemporal beams. Compared to Gaussian beams, structured light beams feature a more complex spatial structure, which can be employed in a wide range of applications. The light beam carrying orbital angular momentum (OAM), a kind of structured light beams, is featured by a helical phase front of exp(i*lθ*), where *l* is the topological charge and *θ* the azimuthal angle [4]. The topological charge *l*, in principle, taking an unlimited value, denotes the twisting rate of the helical phase front, while the handedness of which is represented by the sign of *l*. Due to the helical phase structure, an OAM beam features a doughnut intensity profile with a phase singularity at the beam center. Driven by their distinctive properties, i.e., helical phase structure and doughnut intensity profile, OAM beams have attracted increasing interest in recent years, giving rise to many developments in astronomy, manipulation, microscopy, imaging, metrology, sensing, nonlinear interactions, quantum science, and optical communications [5], [6], [7], [8], [9], [10], [11], [12], [13], [14].

With the arrival of big data era, the dramatic growth of global Internet traffic and myriads of emerging massively data-intensive use applications such as multi-way virtual meeting have fueled ever increasing research efforts for sustainable expansion of network capacity. Beyond various advanced modulation formats and traditional multiplexing techniques, including *m*-ary phase-shift keying (*m*-PSK), *m*-ary quadrature amplitude modulation (*m*-QAM), orthogonal frequency-division multiplexing (OFDM), wavelength-division multiplexing (WDM), optical time-division multiplexing (OTDM), and polarization-division multiplexing (PDM) by exploiting complex amplitude (amplitude, phase), frequency (wavelength), time and polarization physical dimensions of photons [15], [16], [17], [18], space-division multiplexing (SDM) is recognized as an alternative technique to facilitate the effective scaling of transmission capacity by tailoring the spatial structure of photons. OAM beams, having unique properties of inherent orthogonality and unbounded states in principle, have been widely used in SDM-based optical communications [19], [20], [21], [22], [23], [24], [25], [26], [27].

In this paper, we focus on free-space optical communications using OAM and beyond. First, we introduce different light beams carrying OAM and beyond, as well as generation and detection of OAM beams. Then, we show the concept of optical communications using OAM and various types of OAM optical communications. After that, we review recent research progress in OAM modulation, OAM multiplexing, OAM multicasting, OAM communications in turbulence, structured light communications, diverse and robust communications. Finally, prospects and upcoming challenges of OAM communications and beyond are discussed at the end.

## 2 Fundamentals of OAM

### 2.1 Light beams carrying OAM and beyond

**Figure 1:**
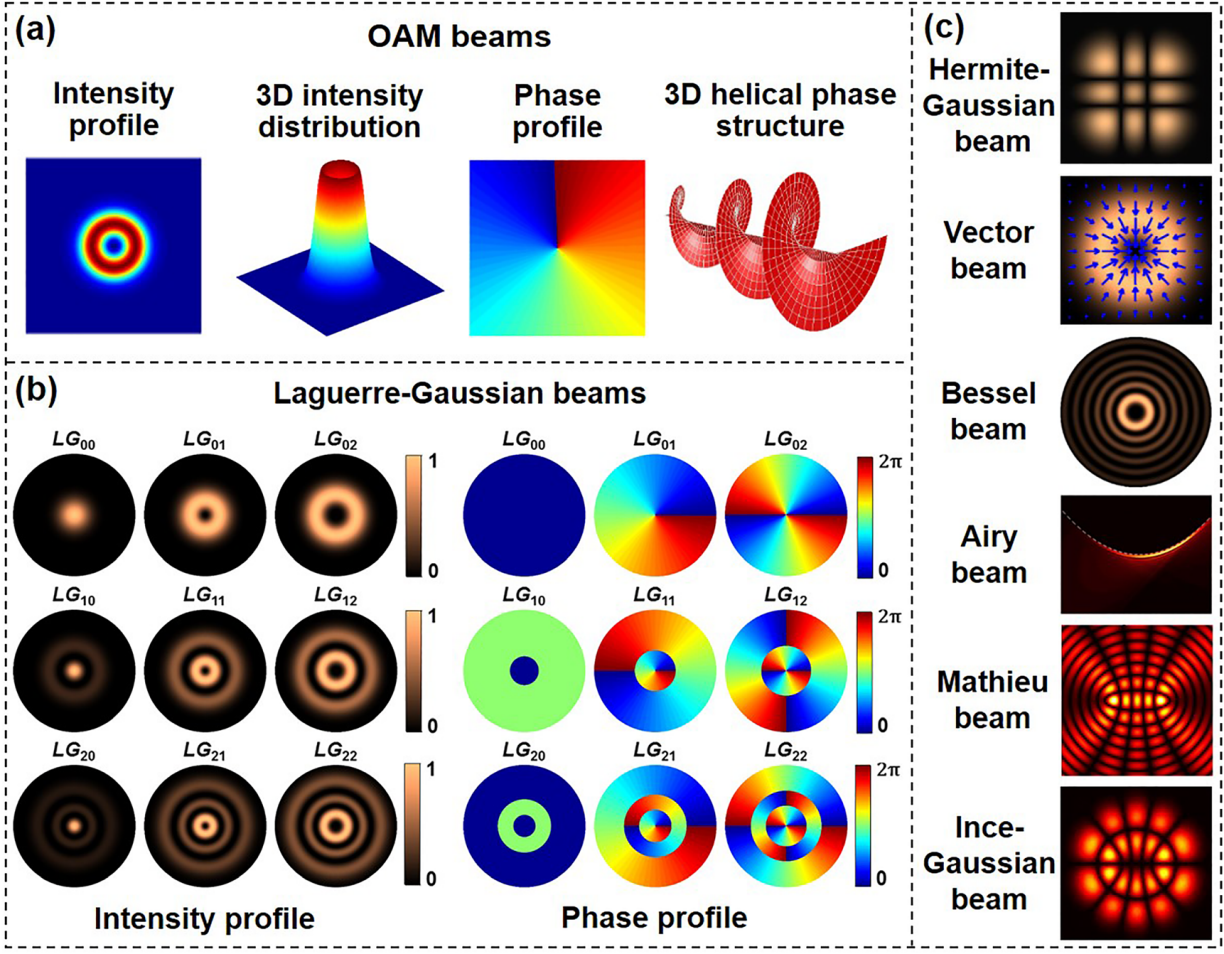
Different types of structured light beams. (a) Intensity profile, 3D intensity distribution, phase profile, 3D helical phase profile of OAM beams; (b) intensity and phase profiles of Laguerre–Gaussian (LG) beams; and (c) other structured light beams: Hermite–Gaussian (HG) beam, vector beam, Bessel beam, Airy beam, Mathieu beam, Ince–Gaussian beam.

As known to all, linear momentum and angular momentum are important physical quantities, collectively called momentum. In fact, spiral phenomena are very familiar in our daily life, for example spiral stairs, snail shell, sunflower seed arrangement, galaxies/nebulae, etc. Similarly, we can also see spiral phenomena in angular momentum. The wavefront of helically-phased light waves features 3D spiral structure and each of their photons carries an OAM. Figure 1a displays the intensity profile, 3D intensity distribution, phase profile, 3D helical phase structure of a typical OAM beam. There are many types of light beams carrying OAM in free space, e.g., Laguerre–Gaussian (LG) beams, Hermite–Gaussian (HG) beams, Bessel beams, Mathieu beams, Ince–Gaussian beams, Helmholtz–Gauss beams, Laplace–Gauss beams, hypergeometric beams, hypergeometric-Gaussian beams, radial carpet beams, and so on [28], [29], [30], [31], [32], [33], [34]. The LG beam is a typical OAM beam which can be described as follows in cylindrical coordinates [35],

$$u\left(r,\varphi ,z\right)=E_{0}{\left[\frac{r\sqrt{2}}{w\left(z\right)}\right]}^{l}L_{m}^{l}\left(\frac{2r^{2}}{w^{2}\left(z\right)}\right)\frac{\omega_{0}}{w\left(z\right)}exp\left[-i\phi_{ml}\left(z\right)\right]exp\left[i\frac{k_{0}r^{2}}{2q\left(z\right)}\right]exp\left(il\varphi \right)$$

where r is radius, φ is azimuthal angle and *z* is propagation direction. $w\left(z\right)=w_{0}{\left[\left(z^{2}+z_{R}^{2}\right)/z_{R}^{2}\right]}^{1/2}$ represents the beam radius at a distance *z* away from the beam waist, in which $w_{0}$ is the radius of the beam waist, $z_{R}$ is the Rayleigh distance. $k_{0}$ represents the wave vector of light beam in vacuum. *q*(*z*) equals to *z*-*iz*_*R*_, $\phi_{ml}\left(z\right)=\left(2m+l+1\right)\text{arctan}\left(z/z_{R}\right)$ is the Gouy phase, and $L_{m}^{l}\left(x\right)$ is generalized Laguerre polynomial. We can see that the intensity profile of LG beams is determined by *m* an *l*, where *m* is the number of rings in the radial direction and *l* is the topological charge of LG beams. Figure 1b shows the simulated intensity and phase profiles of different LG beams with different topological charges. Figure 1c illustrates some other structured light beams such as HG beam, vector beam, Bessel beam, Airy beam, Mathieu beam, and Ince–Gaussian beam.

### 2.2 Generation and detection of OAM beams

**Figure 2:**
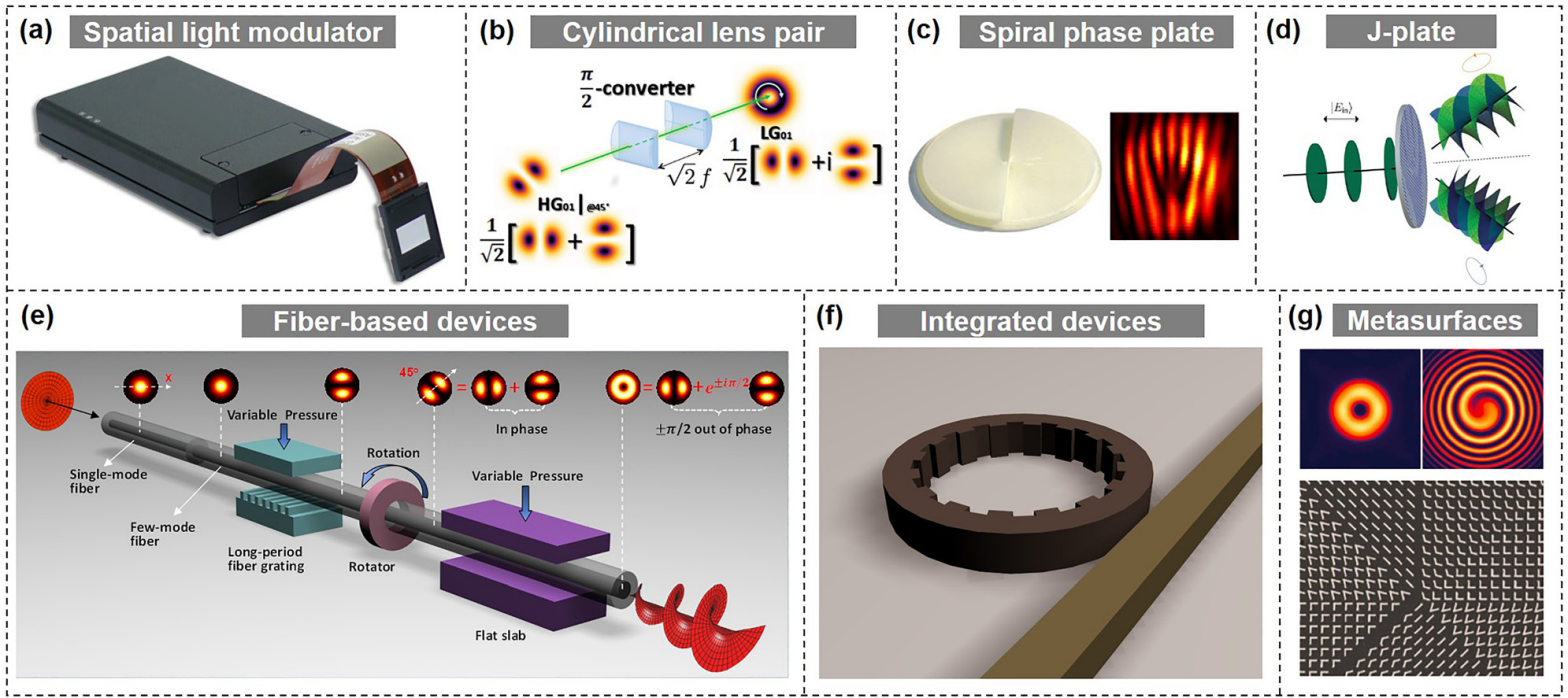
Typical OAM generation techniques (spatial light modulator [SLM] [20, 39], cylindrical lens pair [35], spiral phase plate [45], J-plate [47], fiber-based devices [51], photonic integrated devices [53], metasurfaces [55]). Reprint permission obtained from [20, 35, 39, 45, 47, 51, 53, 55].

OAM beams have been employed in a variety of applications. However, for all these applications, the first and important thing is to generate and detect OAM beams. Various techniques can be used to generate OAM beams, including spatial light modulator (SLM) with diffractive phase pattern/hologram [20, 36], [37], [38], [39], [40], [41], [42], [43], [44], cylindrical lens pairs [35], spiral phase plate (SPP) [45, 46], J-plate [47], fiber-based devices [48-51], photonic integrated devices [52], [53], [54], and metamaterials/metasurfaces [55], [56], [57], as displayed in Figure 2. In general, OAM detection can be realized in an opposite way for most of them. Other OAM detection techniques include interferometry, spatially variable retardation plates, annular gratings, plasmonic photodiodes, and digital coherent receiver [58], [59], [60]. For the OAM (de)multiplexing, the commonly used device is a beam splitter which however introduces additional 3-dB loss and is not scalable. Specially designed complex phase mask and Dammann grating are alternative devices enabling OAM (de)multiplexing with improved performance but still limited scalability [43]. Recently, mode sorters based on optical geometric transformations [61], [62], [63] or photonic integrated circuits advance both efficient OAM multiplexing and demultiplexing with great scalability.

## 3 Concept of optical communications using OAM

Generally speaking, optical communications or more general electromagnetic communications play with different physical dimensions or degrees of freedom of electromagnetic waves to transfer data information. OAM optical communication is also a type of communications using electromagnetic waves to carry and deliver data information.

**Figure 3:**
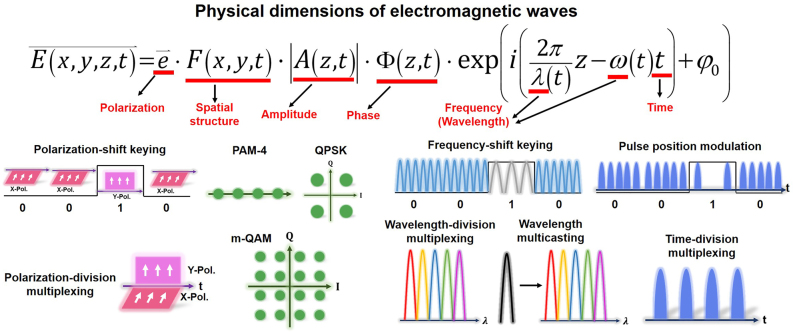
Illustration of physical dimensions/degrees of freedom of electromagnetic waves (frequency/wavelength, amplitude, phase, polarization, time, spatial structure).

Figure 3 illustrates basic physical dimensions or degrees of freedom of electromagnetic waves, including frequency (wavelength), amplitude, phase, polarization, time, and spatial structure. Actually, manipulation of those degrees of freedom in physics enables various kinds of communications. Despite great success of scaling the capacity limits by frequency/amplitude/phase/time/polarization dimensions (e.g., *m*-QAM, OFDM, WDM, OTDM, PDM) in the past decades, further continuous increase of capacity relying on those physical dimensions will be more difficult. Thus, it is highly desired to explore the spatial structure physical dimension of electromagnetic waves. OAM is related to the spatial phase structure physical dimension of photons. Remarkably, one distinct feature of OAM is its unlimited (in principle) and intrinsically orthogonal states, i.e., multiple OAM beams with different topological charge values are inherently orthogonal with each other, which make it possible to develop optical communications using OAM.

**Figure 4:**
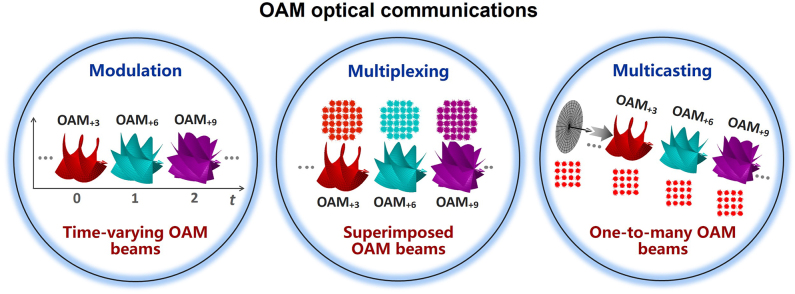
Different types of OAM optical communications (OAM modulation, OAM multiplexing, OAM multicasting).

The typical types of optical communications employing different physical dimensions of photons include modulating, multiplexing and multicasting data information, so does OAM optical communication as shown in Figure 4. OAM modulation refers to encode data information by different OAM beams, where data information is directly encoded by time-varying OAM states. For OAM multiplexing, multi-channel data information modulated by other physical dimensions such as amplitude and phase are distinguishable from each other when carrying different OAM values, i.e., different OAM beams are used as independent carriers to deliver different data information. OAM multicasting duplicates data information into multiple copies (carrying different OAM values) for multiple end users (i.e., one-to-many communications).

## 4 OAM modulation

### 4.1 OAM modulation based on spatial light modulators

**Figure 5:**
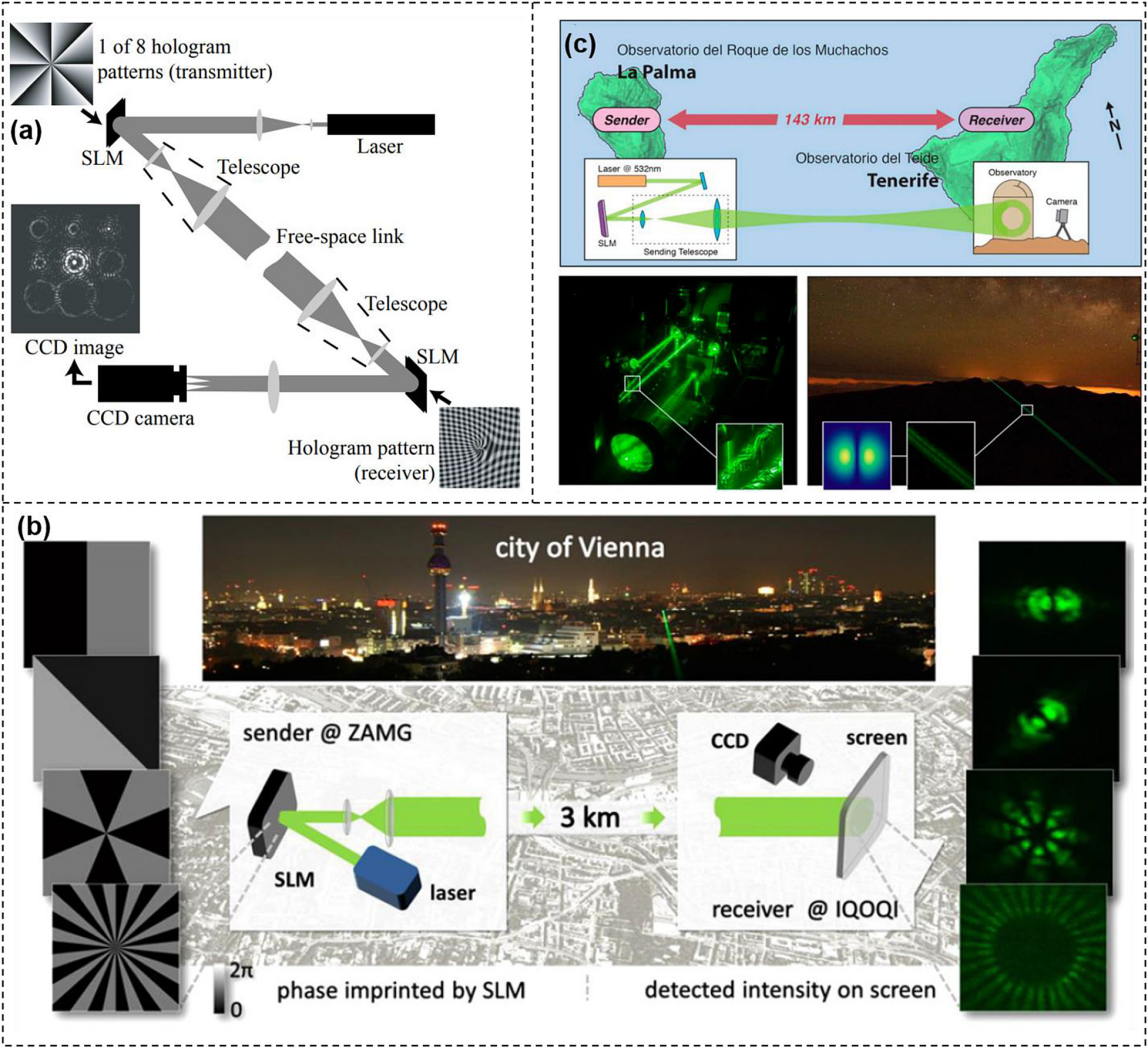
OAM modulation communications using SLM. (a) Experimental setup for free-space OAM coding/decoding communications [64]; (b) experimental setup over 3-km intra-city link in Vienna using OAM superposition modes [65]; (c) experimental setup and scene for free-space 143-km superposed OAM beams coding/decoding communications [66]. Reprint permission obtained from [64–66].

OAM modulation communication was first experimentally demonstrated in free space in 2004 [64]. This experiment demonstrated that the OAM modulation was able to prevent eavesdropping in a 15-m free-space optical link as shown in Figure 5a. The transmitter consisted of a He–Ne laser, an SLM loaded with different phase holograms, and a telescope to expand the beam size. Eight phase holograms were prepared to generate eight different OAM beams corresponding to *l* = −16, −12, −8, −4, 4, 8, 12, 16, which referred to different data information by switching the phase holograms at different time. The receiver was based on a similar telescope to reduce the beam size, another SLM, and a CCD camera. The phase hologram loaded onto the SLM was specially designed to detect the time-varying OAM beams. The hologram was designed to diffract the light beam into nine beams, each with a different topological charge, arranged in a 3 × 3 grid. A CCD was followed to detect the OAM state. The results indicated that OAM could be used to encode data onto a laser beam for transmitting information in free-space optical systems. Later in 2014, OAM superposition modes modulation transmission was demonstrated over 3-km intra-city link in Vienna under strong turbulence conditions [65]. The setup was displayed in Figure 5b. Assisted by a standard adaptive pattern recognition algorithm, an incoherent detection scheme was introduced to measure the clear intensity profiles on a screen. Such scheme avoided coherent phase measurements for detecting the transmitted OAM beams, which would be affected by the atmospheric contribution. Sixteen different superimposed OAM beams were employed in the experiment for modulation transmission with the average error rate reaching to 1.7%. Further, they demonstrated OAM superposition modes modulation transmission over a distance of 143-km between two Canary Islands as shown in Figure 5c [66]. Three superimposed OAM beams were employed to encode data information, which were distinguished by an artificial neural network at the receiver. With the help of the algorithm, the detection accuracy of different OAM superposition modes was more than 80% and the error rate of decoding the modulation information reached to 8.33%.

### 4.2 High-speed OAM modulation

In general, the OAM switching or manipulating devices are SLMs or q-plates in the OAM modulation communication links, which are slow to respond (tens of Hz) [37, 39, 40, 42, 45, 57, 67], [68], [69]. The modulation speed greatly limits the prospect of wide use of OAM modulation communication in practical optical communication systems. In this section, we show some examples of high-speed OAM modulation communications in free space.

**Figure 6:**
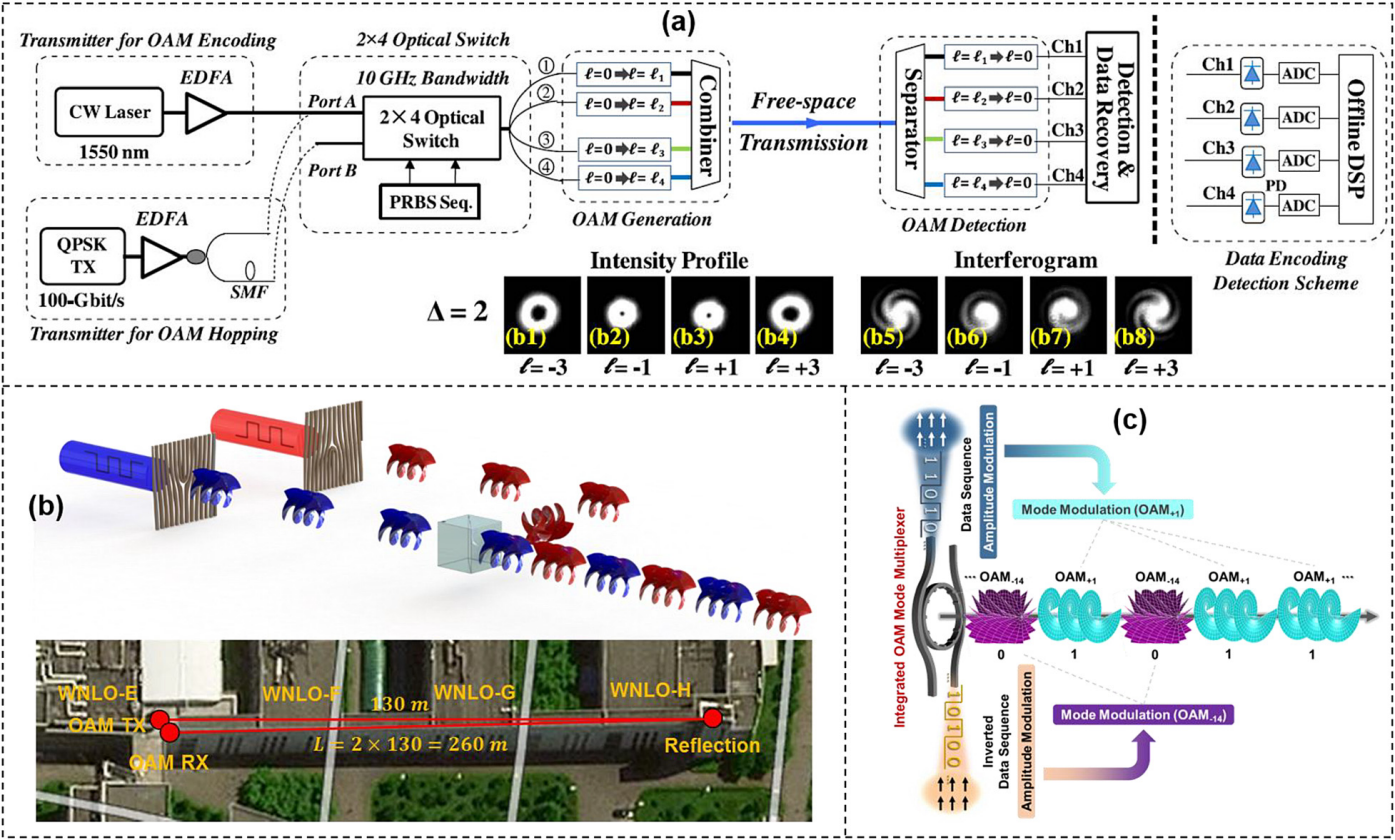
High-speed OAM modulation communications. (a) OAM-based data encoding and hopping systems [70]. (b) Concept and principle of high-speed OAM modulation and layout of a 260-m 25-Gbit/s OAM modulation data transmission link between WNLO-E building and WNLO-H building. WNLO: Wuhan National Laboratory for Optoelectronics. (c) Concept and principle of high-speed photon dimension mapping (amplitude-to-OAM modulation mapping) for OAM modulation assisted by an integrated OAM beam multiplexer. Reprint permission obtained from [70].

In 2015, a high-speed data encoding scheme at 20 Gbit/s using four OAM beams was demonstrated as shown in Figure 6a [70]. The influence of mode spacing and time misalignment between mode channels on the switching crosstalk and bit-error rates (BERs) was investigated. The adjacent modes with a mode spacing of one introduced an extra power penalty of 3.2 dB compared to a larger mode spacing. Besides OAM modulation communications over few meters in the labs, long-distance high-speed OAM modulation communication was also achieved in free space. The concept and principle of high-speed OAM modulation were illustrated in Figure 6b. Two light beams with the same wavelength carrying opposite 25-Gbaud on–off keying (OOK) signals launched onto two SLMs with different holograms. The two beams were combined together by a beam splitter (BS) to map the temporal domain OOK signals to two orthogonal OAM beams with different topological charges. Each OAM beam represented a symbol occupying a 40-ns period. The layout of a 260-m 25-Gbit/s OAM modulation data transmission link between the WNLO-E building and WNLO-H building was shown at the bottom of Figure 6b, which was exposed to the atmospheric conditions. The transmitter and receiver were located at WNLO-E building while the reflection mirror (M) was located at the end of the corridors.

Most of OAM modulation communications employ SLMs to generate OAM beams [71], [72], [73], which are relatively bulky and expensive despite the impressive performance. Photonic integration is clearly the trend and the key enabler toward compact, reliable and low-cost optical devices that are highly desired in optical communications [74], [75], [76], [77], [78], [79]. We demonstrated a 15-Gbit/s OAM beam (OAM_+1_ and OAM_−14_) modulation by employing an integrated OAM beam multiplexer as shown in Figure 6c. Two separate light beams with the same wavelength emitting from two lasers were modulated by two intensity modulators driven by two opposite OOK data sequences from a bit-pattern generator. Then the two light beams were fed into the two bus waveguides I and II of the ring resonator with different widths. The ring resonator waveguide supported two transverse electric modes, i.e., TE_00_ and TE_01_. The widths of the ring resonator waveguide and the two input bus waveguides were engineered in such a way that bus waveguides I and II selectively excited TE_00_ and TE_01_ whispering gallery modes (WGMs) in the ring resonator waveguide at a wavelength near 1550 nm. The excited TE_00_ and TE_01_ WGMs were then coupled to different OAM beams by the angular grating with mode order *l* = *p* − *q* (*p* is the WGM optical periods and *q* is the number of grating elements). By carefully adjusting the length of two light paths, we mapped the Gaussian beam OOK data sequence to a time-varying OAM beam sequence (i.e., OAM_+1_ and OAM_−14_ were encoded to represent 1 and 0, respectively). Thus, we realized high-speed amplitude-to-OAM modulation mapping seeded by an integrated OAM beam multiplexer.

### 4.3 Spatial array modulation

In OAM modulation communications, the amount of bit information carried by one symbol increases with the number of OAM beams *N* used for modulation. The modulation efficiency can be improved Log_2_ *N* times compared to binary encoding. In addition to increase the modulation speed, enlarging the OAM beams set for modulation is another way to increase the capacity of an OAM modulation communication link. Although orthogonal OAM beams are unlimited in principle, the available states may be limited by actual situations, such as generation of OAM beams with large topological charge numbers.

**Figure 7:**
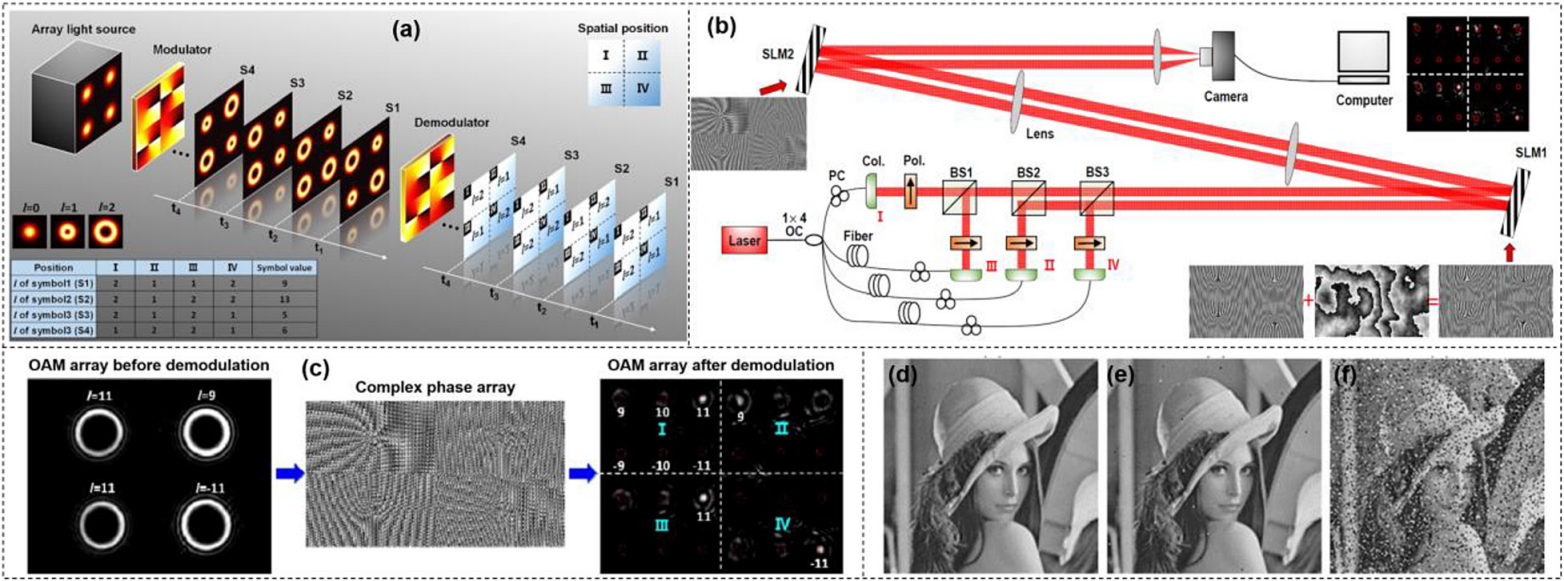
OAM array modulation communications. (a) Concept of OAM modulation with multiple OAM array states for optical interconnects. Several OAM beams were placed in different spatial positions, forming an OAM beam array. The data sequence was encoded to OAM array sequence. Each OAM array state represented a data symbol value. (b) Experimental setup for OAM array modulation with simultaneous multi-OAM demodulation method. OC: optical coupler; PC: polarization controller; Col.: collimator; Pol.: polarizer; BS: beam splitter; SLM: spatial light modulator. (c) The complex phase mask array loaded onto the SLM2 and the intensity profiles of OAM array {11, 9, 11, −11} before and after demodulation. (d) Original image with 256 different grayscale values and 150 × 150 pixels. (e) and (f) recovered image after optical interconnects when the correlation lengths *r*_0_ of turbulence phase masks were 3 and 1 mm, respectively [81]. Reprint permission obtained from [81].

To increase the efficiency of OAM modulation with a few number of OAM beams, we demonstrated a new modulation scheme. Inspired by few-mode multi-core fiber [80], OAM array modulation combining OAM beams and spatial position was shown in Figure 7a [81]. An OAM beam array is composed of serval OAM beams distributing in different spatial positions. One OAM array state represents a symbol code, which can be achieved by changing the topological charge of OAM beams at different spatial positions. The total number of OAM array states is *N* ^*n*^, where *N* is the number of modulating OAM beams in single spatial positions and *n* is the number of spatial positions of OAM array.

**Figure 8:**
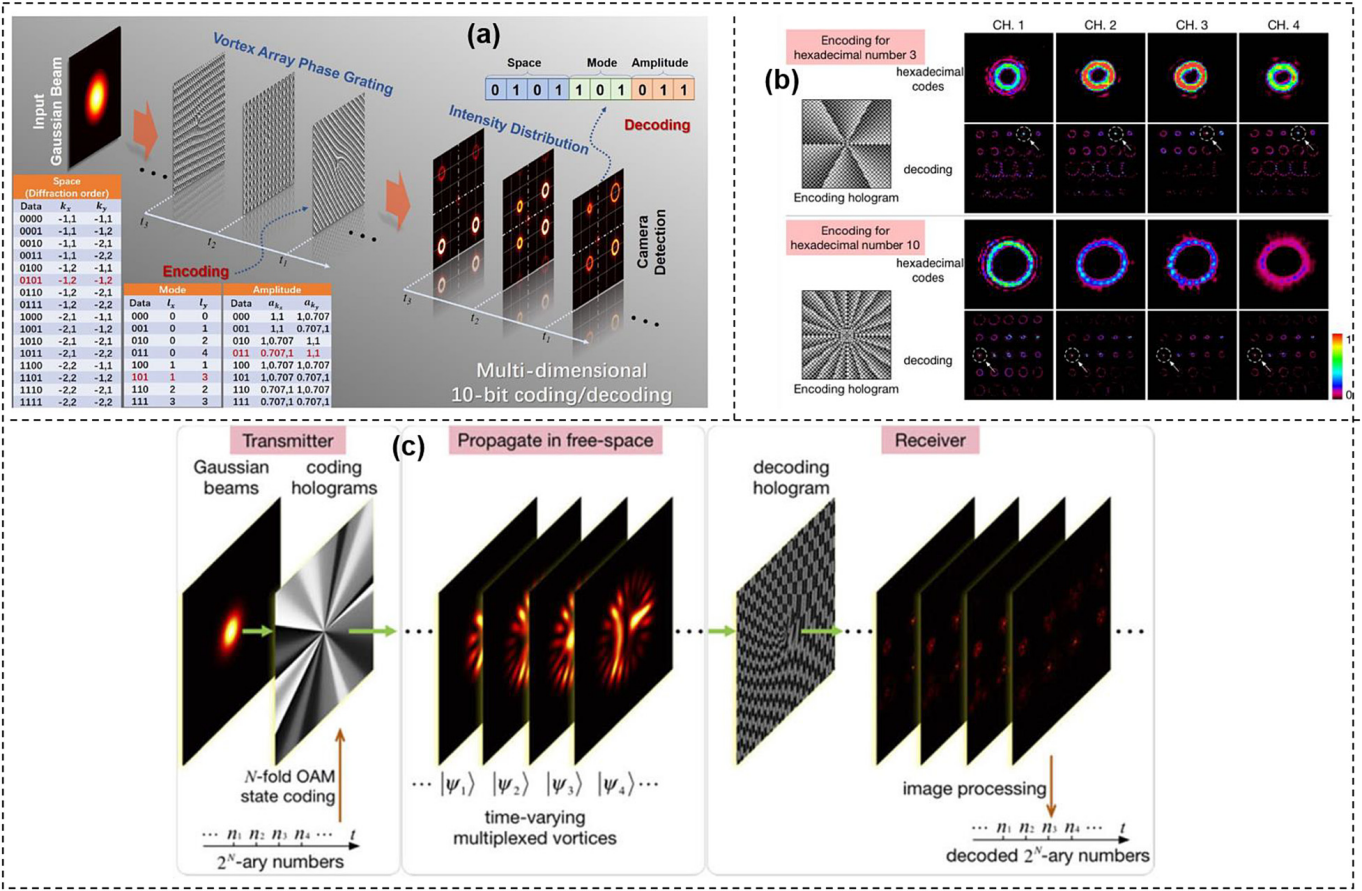
More examples of OAM array modulation communications. (a) The concept and principle of multi-dimensional space/mode/amplitude coding/decoding using the vortex array phase grating. The coding table was shown in the lower-left corner [82]. (b) Hexadecimal codes and their corresponding decoding patterns captured experimentally by each channel in digital signal multicopy replication [83]. (c) Concept of data transmission based on multi-state OAM shift keying [84]. Reprint permission obtained from [82–84].

The experimental setup of OAM array modulation with simultaneous multi-OAM demodulation method was shown in Figure 7b. To form the beam array, the light beam was split into four paths (I, II, III, IV) relatively delayed with fibers. Four collimated Gaussian beams with different horizontal and vertical positions (I and II were above III and IV, I and III were on the left of II and IV) were gathered by three BSs. The Gaussian beams array launched onto SLM1 which was loaded with phase holograms dividing into four parts to generate OAM array sequence, i.e., modulating information onto OAM arrays. Two OAM beam sets {*l* = ±9, ±10, ±11} and {*l* = ±9, ±12, ±15} were employed in the experiment to analyze the effect of different space on the performance of the OAM array modulation link. The complex phase hologram loaded onto SLM2 was used to realize simultaneous multi-OAM demodulation. A CCD camera worked as a detector to measure the intensity profiles of the OAM array and the information was recovered by the measured image through offline processing.

The principle of simultaneous multi-OAM demodulation scheme was shown in Figure 7c. The input OAM array was {*l*_Ⅰ_ = 11, *l*_Ⅱ_ = 9, *l*_Ⅲ_ = 11, *l*_IV_ = −11}. After passing through the complex hologram, the OAM beam array was back converted to Gaussian-like beams and steered to the desired locations. From the measured intensity profiles of OAM array demodulation, the topological charge of each OAM in the OAM array could be easily distinguished by filtering out the central bright spot and discriminating its position. To further evaluate the performance of the OAM array modulation link with turbulence, OAM array set {*l* = ±9, ±12, ±15} was used for modulation transmission of a grayscale image. Figure 7d displayed the original image with 150 × 150 pixels having 256 different grayscale values. Each pixel could be represented by 8 bit, thus the total number bits of the image was 150 × 150 × 8 = 180,000. After OAM array modulation, the symbol length was 16,929 which was reduced by 10.63 times compared to the original binary data. Figure 7e and f displayed the recovered images with BER about 1.2e-3 and 0.14, corresponding to the correlation lengths *r*_0_ of turbulence phase masks equaling to 3 and 1 mm, respectively.

To make the OAM array generation more flexible, another design to construct vortex array phase grating (VAPG) was introduced as shown in Figure 8a [82]. The proposed VAPG was able to realize multi-dimensional modulation including space position, OAM beams and amplitude. Vortex array with different mode states and relative power could be generated by changing the parameters of VAPGs. In addition, a 10-bit multi-dimensional data modulation scheme for image transfer in a free-space link was experimentally demonstrated with a zero BER, which confirmed the feasibility of the proposed VAPG-based modulation scheme. Figure 8b proposed an OAM modulation based free-space one-to-many communication link [83]. At the transmitter, the multiple OAM encoding was realized by loading a series of specially designed holograms to SLM, transforming Gaussian beams into four groups of time-varying OAM beams with various directions, simultaneously. The transmitted four groups of signals were captured and demodulated by four receivers separately. In addition to multicopy replication of the same digital signal, various signal one-to-many communication was also accomplished, where four gray images were modulated by one transmitter simultaneously and sent separately to four different receivers. Figure 8c demonstrated a multi-mode OAM shift keying in free space through single hologram over 10 m [84]. In the proposed scheme, the modulation was done by switching a series of holograms to get a time-varying OAM sequence. After free-space transmission, the OAM sequence was demodulated by a Dammann grating and analyzed by the image processing. As a proof of concept, 8 bits multi-state OAM shift keying through 8-fold multiplexed OAM beams was achieved in free space over 10 m.

## 5 OAM multiplexing

### 5.1 Ultra-high spectral efficiency

In addition to the ability to boost the transmission capacity, one more benefit from OAM multiplexing is the efficient use of limited frequency bandwidth resources. By using 16-QAM signals over pol-muxed eight OAM beams in two groups of concentric rings (32 channels in total), we proposed a scheme achieving 95.7 bit/s/Hz spectral efficiency [12]. It is worth mentioning that the four SLMs and lots of accessorial components were used in this experiment, making the setup extremely complicated and limiting its scalability. So, we presented a scheme to facilitate ultra-high spectral efficiency free-space data transmission link with a simplified but still scalable setup, in which only two SLMs were employed to generate OAM beams in the experiment. Specifically, each SLM was loaded with a specially-designed complex phase pattern to generate 11 OAM beams simultaneously, then the 22 OAM beams generated by two SLMs were multiplexed together [85]. Moreover, the spectral efficiency was further expended by incorporating PDM and using high-base modulation format, i.e., OFDM 64-QAM signals.

**Figure 9:**
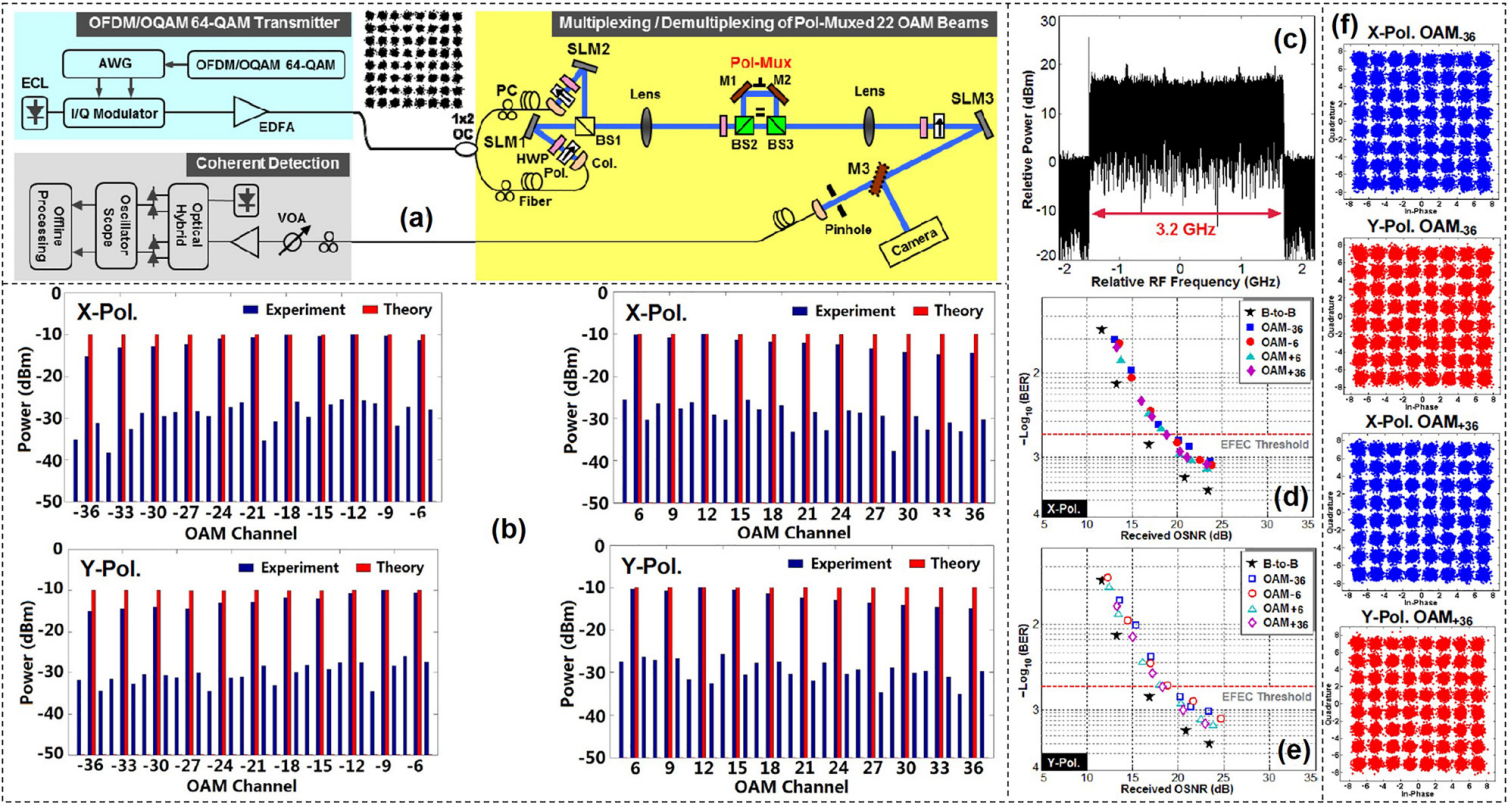
OAM multiplexing communications with a spectral effciency of 230 bit/s/Hz. (a) Experimental setup for OAM multiplexing/demultiplexing (OFDM/OQAM 64-QAM signals over pol-muxed 22 OAM beams). ECL: external cavity laser, AWG: arbitrary waveform generator, EDFA: erbium-doped fiber amplifier, OC: optical coupler, PC: polarization controller, SLM: spatial light modulator, Col.: collimator, Pol.: polarizer, HWP: half-wave plate, BS1: non-polarizing beam splitter, M1-M3: mirror, BS2-BS3: polarizing beam splitter, VOA: variable optical attenuator. (b) Measured power distribution over pol-muxed 22 OAM beams (44 channels in total). (c) RF spectrum of demodulated signal. BER curves for (d) X- and (e)Y-polarized OAM_−36_, OAM_−6_, OAM_+6_, OAM_+36_ channels. (f) Measured constellations for X-polarized (X-Pol.) OAM_−36_, Y-polarized (Y-Pol.) OAM_−36_, X-Pol. OAM_+36_, Y-Pol. OAM_+36_ [85]. Reprint permission obtained from [85].

In the experiment, we achieved an ultra-high spectral efficiency of 230 bit/s/Hz when considering 17.9-Gbit/s OFDM offset QAM (OFDM/OQAM) 64-QAM signals (bandwidth: 3.2 GHz) over pol-muxed 22 OAM beams (44 channels) and including the 7% forward error correction (FEC) overhead. The experiment setup was illustrated in Figure 9a, which consisted of three major parts, i.e., signal transmitter, multiplexing/demultiplexing, and coherent detection. Twenty two OAM beams (±6, ±9, ±12, ±15, ±18, ±21, ±24, ±27, ±30, ±33, ±36) were generated by SLM1 and SLM2 loaded with complex phase holograms. After combined by a BS, the multiplexed OAM beams passed through a polarization multiplexing stage. A half-wave plate (HWP) and a polarizer were used to demultiplex polarization while another SLM3 was used for OAM demultiplexing by switching different holograms. First, we evaluated the performance of the pol-muxed 22 OAM beams by measuring the power distribution over all OAM channels as displayed in Figure 9b. The observed extinction ratio for all pol-muxed 22 OAM channels was larger than 14.1 dB.

Then we measured the performance of 17.9-Gbit/s OFDM/OQAM 64-QAM signals over pol-muxed 22 OAM beams (44 channels in total). The measured radio frequency (RF) spectrum with a bandwidth equaling to 3.2 GHz was shown in Figure 9c. The BER curves of X- and Y-polarized OAM_−36_, OAM_−6_, OAM_+6_, OAM_+36_ channels were displayed in Figure 9d and e, respectively. The observed optical signal-to-noise ratio (OSNR) penalties were less than 3.5 dB at a BER of 2e-3 (enhanced forward error correction (EFEC) threshold). The typical constellations of 64-QAM signals carried by *x*-polarization (X-Pol.) and *y*-polarization (Y-Pol.) OAM_-__36_/_+36_ modes were also shown in Figure 9f.

**Figure 10:**
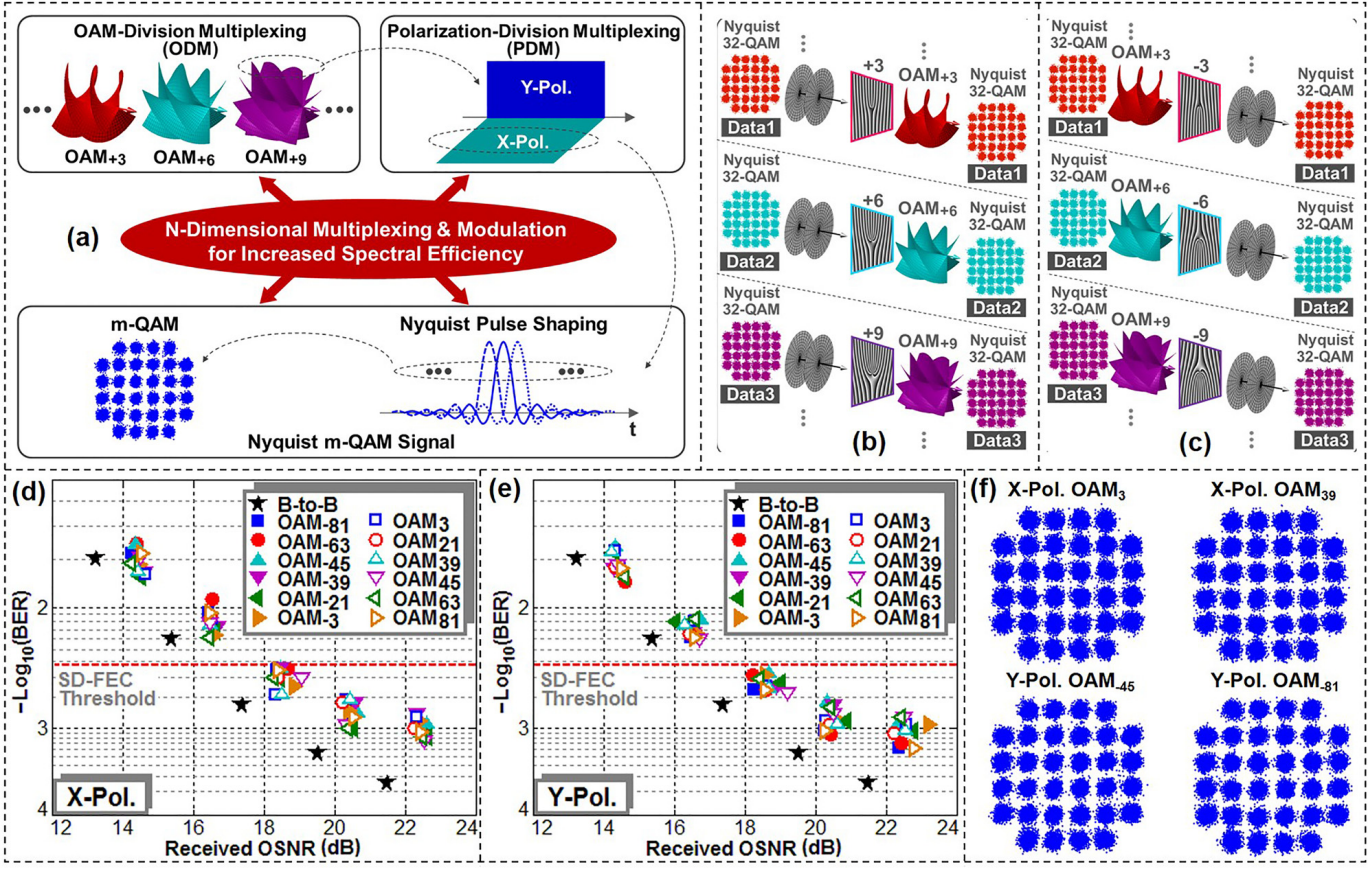
*N*-dimensional OAM multiplexing communications with an ultra-high spectral efficiency of 435 bit/s/Hz. (a) Concept of *N*-dimensional multiplexing and modulation. (b) Conversion from data-carrying Gaussian beams (planar phase front) to data-carrying OAM beams (helical phase front). (c) Back-conversion from data-carrying OAM beams (helical phase front) to data-carrying Gaussian-like beams (planar phase front). Measured BER versus OSNR for typical (d) X- and (e) Y-polarized OAM_−81_, OAM_−63_, OAM_−45_, OAM_−39_, OAM_−21_, OAM_−3_, OAM_3_, OAM_21_, OAM_39_, OAM_45_, OAM_63_ and OAM_81_ channels. (f) Measured constellations for X-polarized (X-Pol.) OAM_3_, X-Pol. OAM_39_, Y-polarized (Y-Pol.) OAM_−45_, Y-Pol. OAM_−81_ [86]. Reprint permission obtained from [86].

To increase the transmission capacity and spectral efficiency of future optical communication systems to a higher degree, employing more channels is a straightforward strategy. Hence, we further presented an improved approach using *N*-dimensional multiplexing together with advanced modulation format signals, i.e., 5.8-Gaud Nyquist 32-QAM signals over pol-muxed 52 OAM beams (104 channels in total) [86]. With the assist of these advanced techniques, we experimentally demonstrated a free-space data transmission link with a total net transmission capacity of 8.16 Tbit/s and an aggregate ultra-high spectral efficiency of 435 bit/s/Hz. The concept of *N*-dimensional multiplexing and modulation was shown in Figure 10a. By combining multiple physical dimensions of photons to modulate and multiplex data information, i.e., OAM-division multiplexing, PDM, Nyquist *m*-QAM signal, the spectral efficiency could be greatly improved by delivering Nyquist *m*-QAM signals over pol-muxed multiple OAM beams. The conversion from data-carrying (e.g., Nyquist 32-QAM) Gaussian beams to data-carrying OAM beams and their back conversion for OAM-division multiplexing using specially designed phase masks were shown in Figure 10b and c.

The experimental setup was similar to the setup in Figure 9a with a Nyquist 32-QAM transmitter and two more SLMs to multiplex more OAM beams. The BER performance of 5.8 Gbaud Nyquist 32-QAM signals over pol-muxed 52 OAM beams at a wavelength of 1550.0 nm was shown in Figure 10d–f. The measured BER curves for X- and Y-polarized typical OAM beams were depicted in Figure 10d and e. The OSNR penalties were less than 2.5 dB at a BER of 3.8 × 10^−3^ (7% soft-decision forward error correction (SD-FEC) threshold). Figure 10f depicted measured typical constellations of Nyquist 32-QAM for X-polarized OAM_3_, X-polarized OAM_39_, Y-polarized OAM_−45_, and Y-polarized OAM_−81_, respectively.

### 5.2 1.036 Pbit/s aggregate capacity and 112.6 bit/s/Hz spectral efficiency

Since OAM multiplexing is also compatible with WDM, we demonstrated a free-space data link with an aggregate transmission capacity of 1.036 Pbit/s and a high spectral efficiency of 112.6 bit/s/Hz by exploiting *N*-dimensional multiplexing, i.e., 54.139-Gbit/s OFDM-8QAM signals over 368 WDM pol-muxed 26 OAM modes [87]. 368 WDM (C + L bands) OFDM-8QAM signals with a total net rate of 19.923-Tbit/s (368 × 54.139 Gbit/s) considering the 20% hard-decision forward error correction (HD-FEC) limit were prepared at the WDM OFDM-8QAM transmitter. 26 OAM beams (±6, ±9, ±12, ±15, ±18, ±21, ±24, ±27, ±30, ±33, ±36, ±39, ±42) were multiplexed in the experiment.

**Figure 11:**
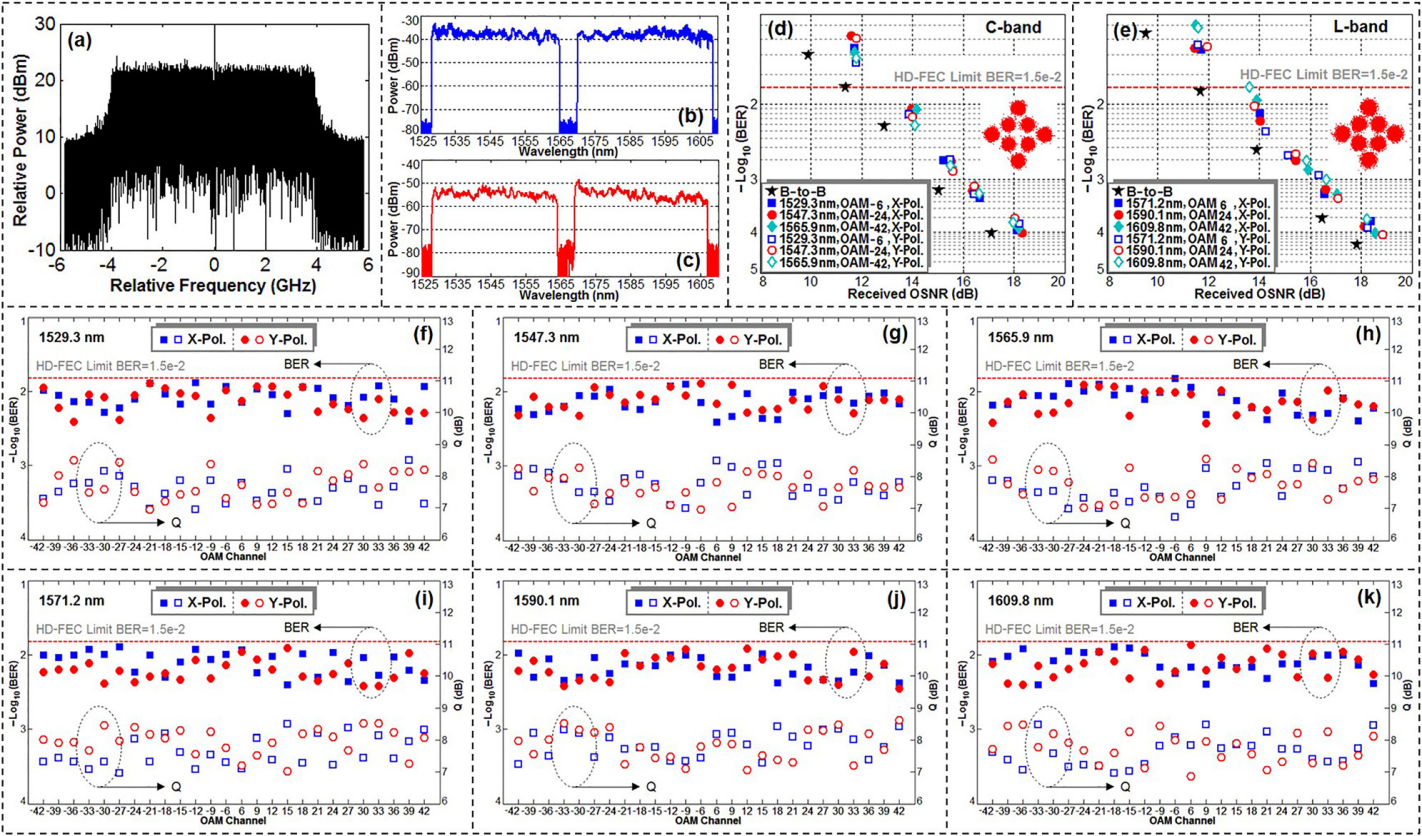
*N*-dimensional OAM multiplexing communications with a transmission capacity of 1.036 Pbit/s. (a) RF spectrum of demodulated signal. Spectra (b) before and (c) after OAM link. BER versus OSNR for single wavelength in (d) C-band and (e) L-band. BER/Q performance of (f) 1529.3 nm, (g) 1547.3 nm, (h) 1565.9 nm in the C-band, (i) 1571.2 nm, (j) 1590.1 nm, (k) 1609.8 nm in the L-band over all pol-muxed 26 OAM beams [87]. Reprint permission obtained from [87].

We then evaluated the performance of 54.139-Gbit/s OFDM-8QAM signals over 368 WDM pol-muxed 26 OAM beams. The RF spectrum of the demodulated signal was shown in Figure 11a. Figure 11b and c plotted measured optical spectra (C + L bands) before and after the OAM transmission link, respectively. The BER curves for typical single wavelength in the C-band and L-band were plotted in Figure 11d and e, respectively. The observed OSNR penalties were less than 2 dB at a BER of 1.5e-2 (HD-FEC limit). Insets of Figure 11d and e depicted measured constellations.

We further comprehensively assessed the performance of *N*-dimensional multiplexing. By selecting 6 wavelengths (1529.3 nm, 1547.3 nm, 1565.9 nm in the C-band, 1571.2 nm, 1590.1 nm, 1609.8 nm in the L-band) out of 368 WDM channels, we measured the BER/*Q* values over all pol-muxed 26 OAM beams as shown in Figure 11f–k. The obtained results showed impressive performance.

### 5.3 Real-world long-distance application scenario

The previous works mentioned above with impressive performance were all demonstrated in the range of few meters in the lab, which ignored the influence of OAM resulting from the real atmospheric turbulence. In real-world scenario application, inhomogeneity of the transmission medium and its fluctuation, i.e., the pressure and temperature variance or the dust in the atmosphere results in variations of the refractive index along the transmission path, which can degrade the performance of free-space optical link, especially for OAM multiplexing communication link. So, it is vital to validate the performance of free-space optical data transmission link.

**Figure 12:**
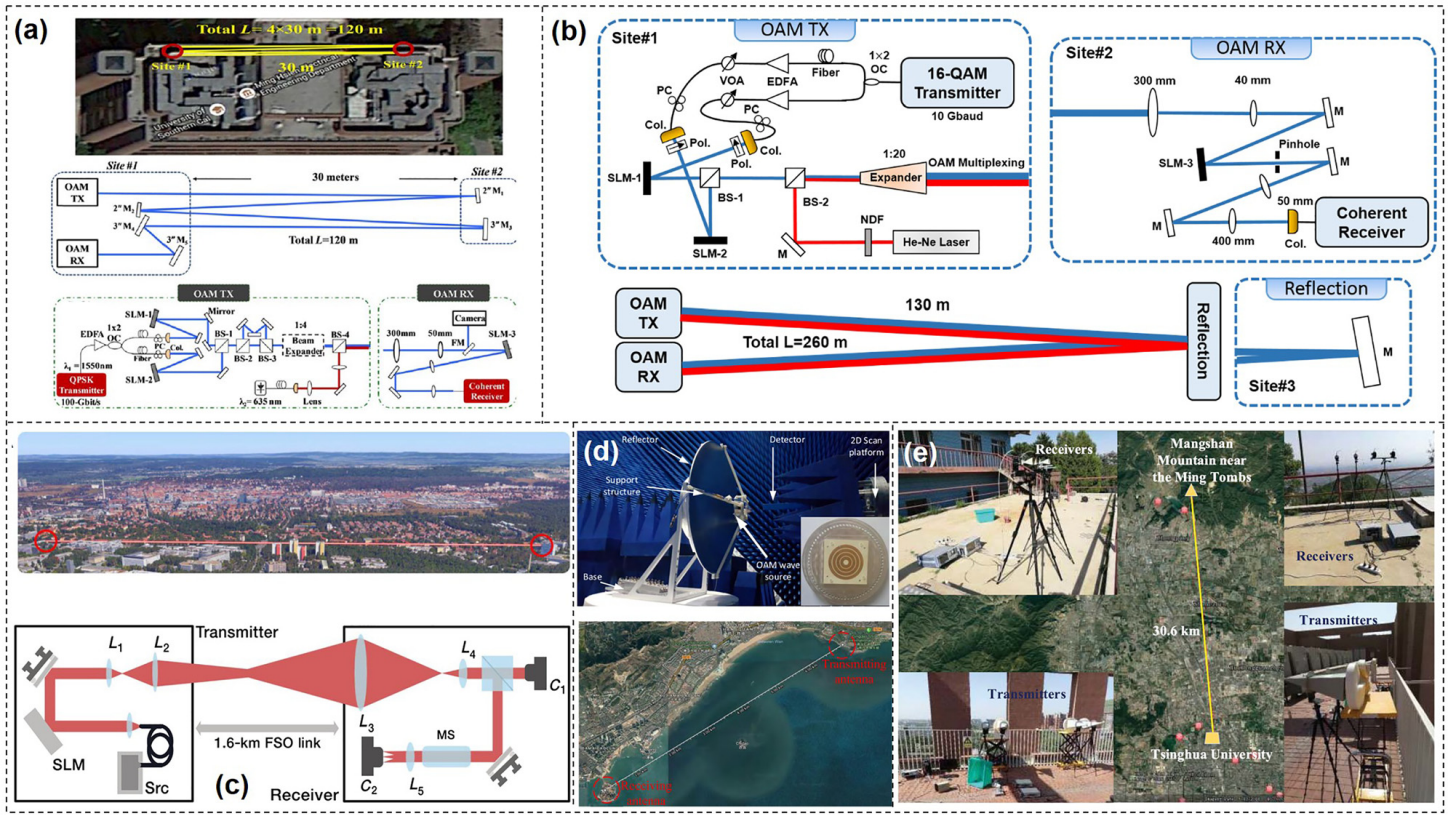
Real-world long-distance application scenario. (a) Experimental setup of a 120-m OAM-multiplexed link on a building roof [88]. (b) Experimental setup of a 260-m security OAM-multiplexed link [89]. (c) 1.6-km free-space link over the city of Erlangen in Germany [90]. (d) Structure of the OAM wave source on the parabolic reflector for long-distance transmission [91]. (e) OAM nondegenerate index mapping for long-distance transmission [92]. Reprint permission obtained from [88–92].

To achieve high-capacity data transmission beyond lab-scale distances based on OAM multiplexing, the performance of a 400-Gbit/s OAM multiplexed free-space optical link over 120 m on the roof of a building was evaluated [88]. Four OAM beams, each carrying a 100-Gbit/s quadrature phase-shift keying (QPSK) signal channel were multiplexed and transmitted. Figure 12a showed the satellite image of the roof and the experimental setup. Furthermore, the influence of channel impairments on the received power, intermodal crosstalk, and system power penalties were investigated. We presented a 260-m security free-space optical data transmission using spatial multiplexing of two OAM beams, where each channel was modulated with 40-Gbit/s 16-QAM data signal [89]. The OAM link performance after 260-m propagation, i.e., beam wandering, received power fluctuation, channel crosstalk, was evaluated in this experiment. The experimental setup was shown in Figure 12b. Two OAM beams with opposite states were multiplexed together (*l* = ±3). The red Gaussian beam produced by a He–Ne laser and combined with the two OAM channels was mainly used for easy system alignment. At the receiving end, the OAM beam was demodulated and coupled into a single-mode fiber (SMF) for coherent detection assisted by off-line digital signal processing. Figure 12c displayed the propagation of a set of orthogonal spatial modes across a free-space channel between two buildings separated by 1.6 km [90]. The preservation of phase purity after transmitting in a real urban environment was evaluated, which was vital for spatial multiplexing. Compared to the theoretical results, the experimental results indicated that adaptations to channel models were required to simulate the effects of atmospheric turbulence placed on high-dimensional structured modes that propagated over a long distance. Thus, with mitigation of vortex splitting, potentially through pre-correction techniques, one could overcome the challenges in a real point-to-point free-space channel in an urban environment. In addition, OAM multiplexing at radio frequency was also a potential candidate. Figure 12d and e demonstrated the OAM transmission across the Yellow Sea with 7 and 30.6 km at radio frequency (RF), respectively [91, 92].

## 6 OAM multicasting

### 6.1 Power-equalized and adaptive power-controllable OAM multicasting

OAM multicasting duplicates data information onto multiple channels having different OAM values, which are orthogonal to each other and could represent different users in a multi-user communication system [93].

**Figure 13:**
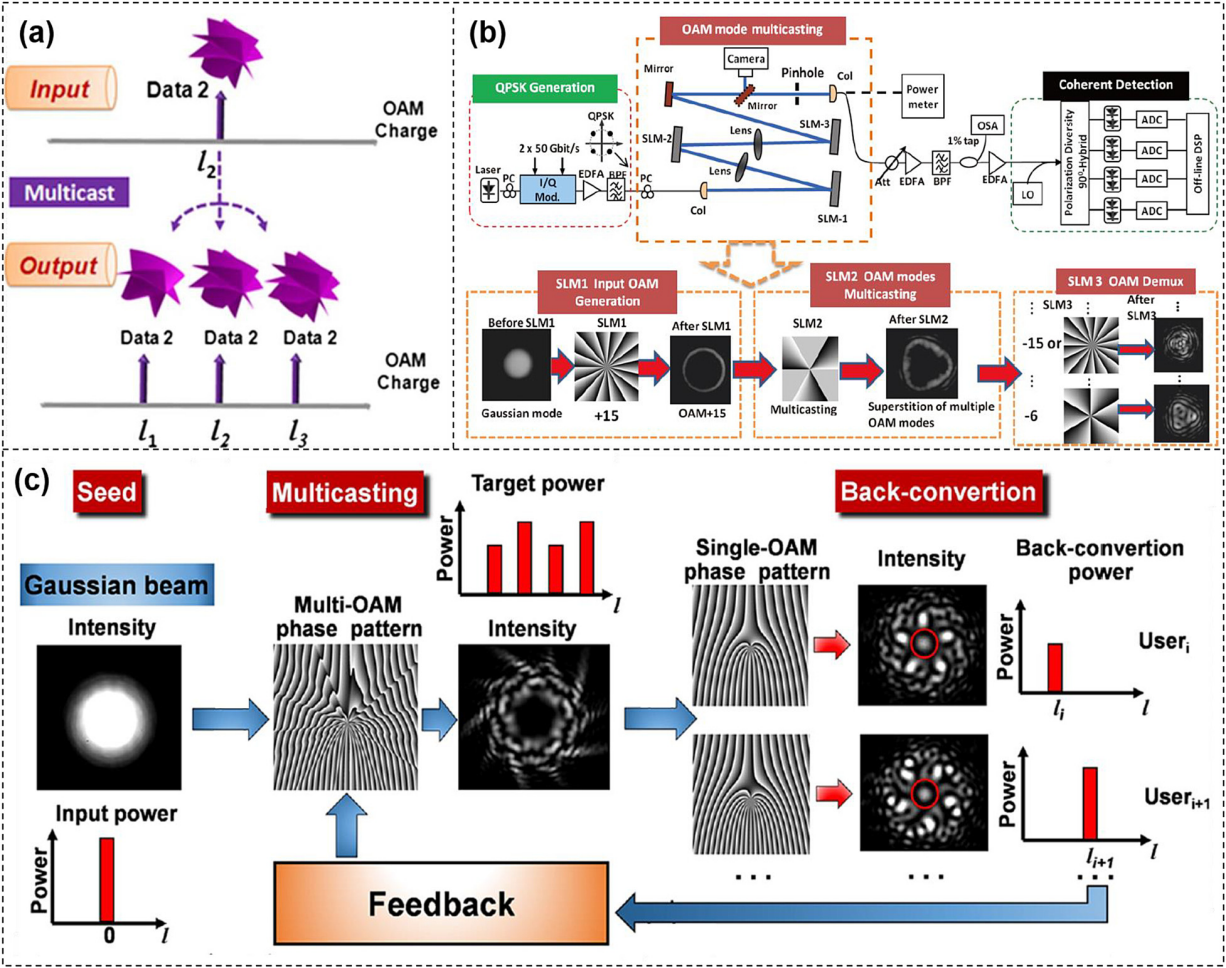
Power-equalized and adaptive power-controllable OAM multicasting. (a) Concept of OAM multicasting [96]. (b) Power-equalized spatial-mode multicasting of a single 100-Gbit/s QPSK carrying OAM beam onto multiple OAM beams [97]. (c) Concept of feedback-assisted adaptive power-controllable OAM multicasting [98]. Reprint permission obtained from [96–98].

Slicing the phase hologram is one of the ways to realize the OAM multicasting function. An angular amplitude aperture of central angle *θ*_c_ with *N*-fold rotational symmetry can distribute energy from the input OAM beam of charge *l* to multiple OAM beams having equally spaced OAM charge number of …, *−kN + l*; …; *−N + l*; *l*; *l + N*; …; *l + kN*; … (*k* is an integer) [94, 95]. The pattern could be designed so that the multicasting channels had almost equalized power without power loss as shown in Figure 13a. By changing the rotational symmetry number *N* and the numbers of the sub-sliced patterns, this approach could be used to multicast more equally spaced OAM channels with a controllable OAM charge number spacing Δ*l* [96]. Figure 13b demonstrated spatial-mode multicasting of a single 100-Gbit/s (50-Gbaud QPSK) OAM beam onto multiple OAM beams [97]. Up to eight multicasted modes were experimentally demonstrated with equalized power and <−20 dB crosstalk. In the multicasting system, the actually measured back-conversion (from OAM beam to Gaussian-like mode) power distribution of the multicast OAM channels is different from each other. This is because different OAM beams feature different back-conversion spot sizes. Figure 13c demonstrated an adaptive power-controllable OAM multicasting scheme by introducing a feedback process [98]. The power of each multicast OAM channel could be arbitrarily controlled by designing and optimizing the complex phase pattern through the adaptive correction of feedback coefficients.

### 6.2 *N*-dimensional 1-to-1100 multicasting

**Figure 14:**
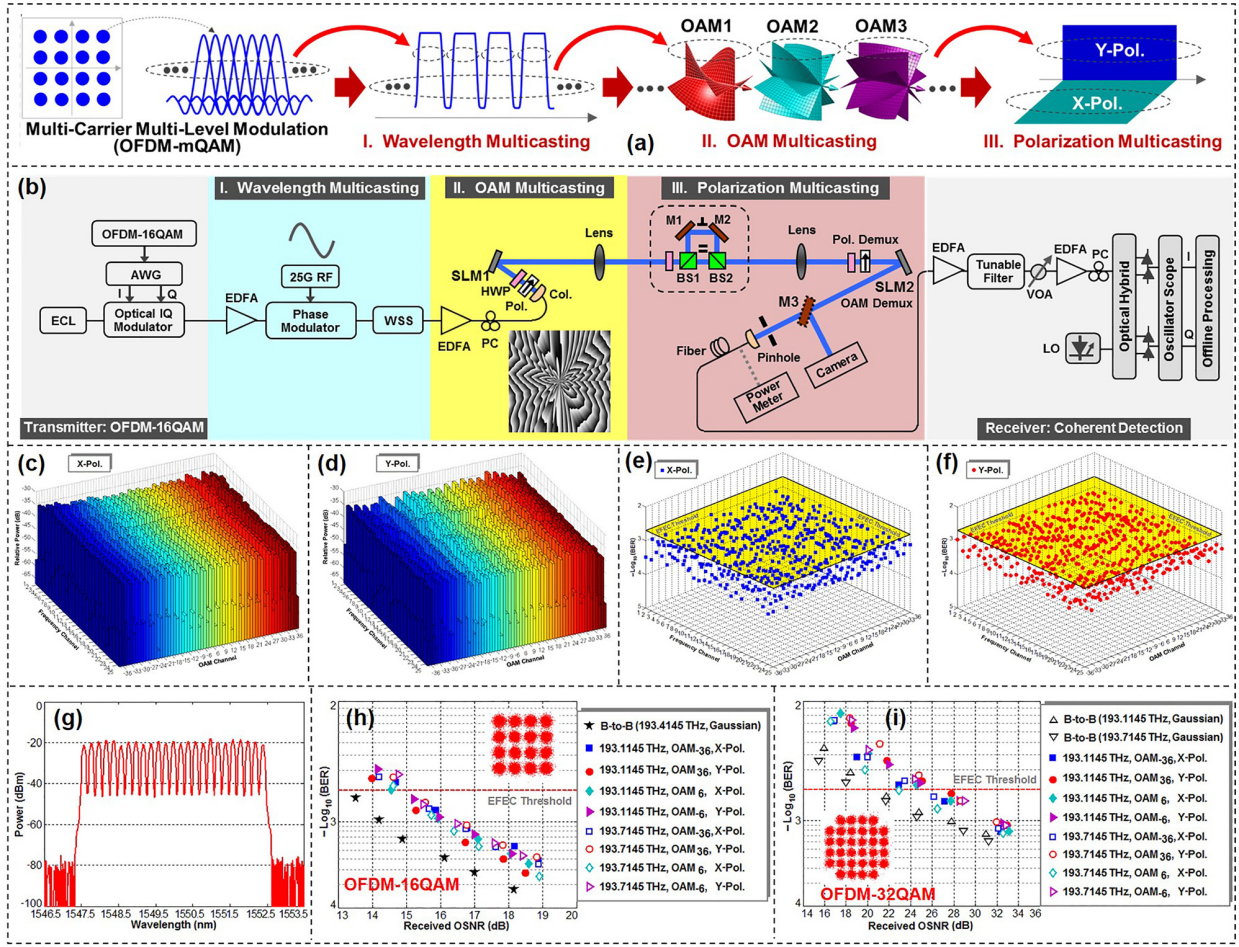
*N*-dimensional 1-to-1100 multicasting. (a) Concept and (b) setup of *N*-dimensional multicasting (wavelength/frequency, OAM, polarization). Inset of (b) showed complex phase pattern. ECL: external cavity laser, PC: polarization controller, AWG: arbitrary waveform generator, EDFA: erbium-doped fiber amplifier, WSS: wavelength selective switch, RF: radio frequency, SLM: spatial light modulator, Col.: collimator, Pol.: polarizer, HWP: half-wave plate, BS: non-polarizing beam splitter, PBS: polarizing beam splitter, BPF: bandpass filter, VOA: variable optical attenuator, LO: local oscillator. (c) and (d) Measured power distributions over all 25 wavelengths, 22 OAM beams and two polarizations (1100 channels in total). (e) and (f) Measured BER performance for all 1100-fold multicasted channels (25 wavelengths × 22 OAM beams × two polarizations). Measured (g) spectrum of 25-fold wavelength multicasting and BER curves for (h) OFDM-16QAM and (i) OFDM-32QAM *N*-dimensional multicasting [99]. Reprint permission obtained from [99].

Assisted by the OAM beams, the multicasting channel could be extended to tens of copies. However, it would be challenge to get an even large number of multicasting copies by utilizing a single physical dimension of photons, e.g., wavelength only or spatial mode only. On the contrast, the combination of multiple physical dimensions would increase the multicasting channel number dramatically. We proposed and demonstrated *N*-dimensional multicasting by combining wavelength/frequency, OAM and polarization physical dimensions. By delivering the OFDM-mQAM signal onto 25 wavelengths each having 22 OAM beams with two polarization states, *N*-dimensional 1-to-1100 multicasting was obtained in the experiment. The concept of *N*-dimensional multicasting was illustrated in Figure 14a [99]. A multi-carrier multi-level modulation (e.g., OFDM-mQAM) signal was duplicated onto multiple wavelengths, multiple OAM beams, and two polarization states in succession. As shown in Figure 14b, the OFDM-MQAM signal was generated at the transmitter which was multicasted in *N* physical dimensions. Wavelength multicasting was carried out by using a phase modulator driven by a 25-GHz sinusoidal RF signal. Followed by a wavelength selective switch (WSS), the power of 25 copies of wavelengths from 193.1145 to 193.7145 THz was equalized. A specially-designed phase hologram loaded onto the SLM completed the multicasting from a single Gaussian mode to 22 OAM beams. The polarization multicasting configuration was formed by two polarizing beam splitters (BS1, BS2) and two mirrors (M1, M2). Finally, by combining wavelength/frequency, OAM and polarization, *N*-dimensional 1-to-1100 multicasting was achieved. At the receiver, the performance of every multicasting channel was measured by polarization demultiplexing, OAM demultiplexing, wavelength filtering, and coherent detection.

Figure 14c and d displayed the measured power spectrum of the entire 1100 multicasting channels, including 25 wavelengths, 22 OAM beams and two polarizations. The measured extinction ratio for all OAM beams was larger than 15.0 dB, which was defined by the power ratio of the desired OAM beam to its left and right neighboring OAM beams. We further measured the BER performance of all 1100-fold multicasted channels. As shown in Figure 14e and f, one could clearly see that all 1100-fold multicasted channels achieved BER <2e-3, indicating the successful implementation of *N*-dimensional 1-to-1100 multicasting. Figure 14g depicted the spectrum of 25 multicasting wavelength channels. Furthermore, Figure 14h and i depicted the BER performance of typical multicasting channels among all the *N*-dimensional 1100 channels carrying OFDM-16QAM signal and OFDM-32QAM signal, respectively.

## 7 OAM communications in turbulence

A critical challenge for free-space optical communications based on OAM is the atmospheric turbulence [100, 101]. The turbulence distorts the wavefront of the OAM beam due to random variations of refractive index of air which is caused by random variations in temperature and convective motion. This kind of distortion increases the inter-model crosstalk between different OAM beams and displacement of the OAM beam, which makes it difficult for OAM detection [102], [103], [104], [105], [106], [107], [108]. In order to mitigate turbulence effects and improve the performance of OAM-based free-space optical communication links, many approaches have been proposed and demonstrated. Generally speaking, typical turbulence mitigation techniques include adaptive optics and digital signal processing to compensate phase distortion caused inter-model crosstalk, and auto-alignment system to compensate scintillation and beam wandering.

### 7.1 Compensation based on adaptive optics

**Figure 15:**
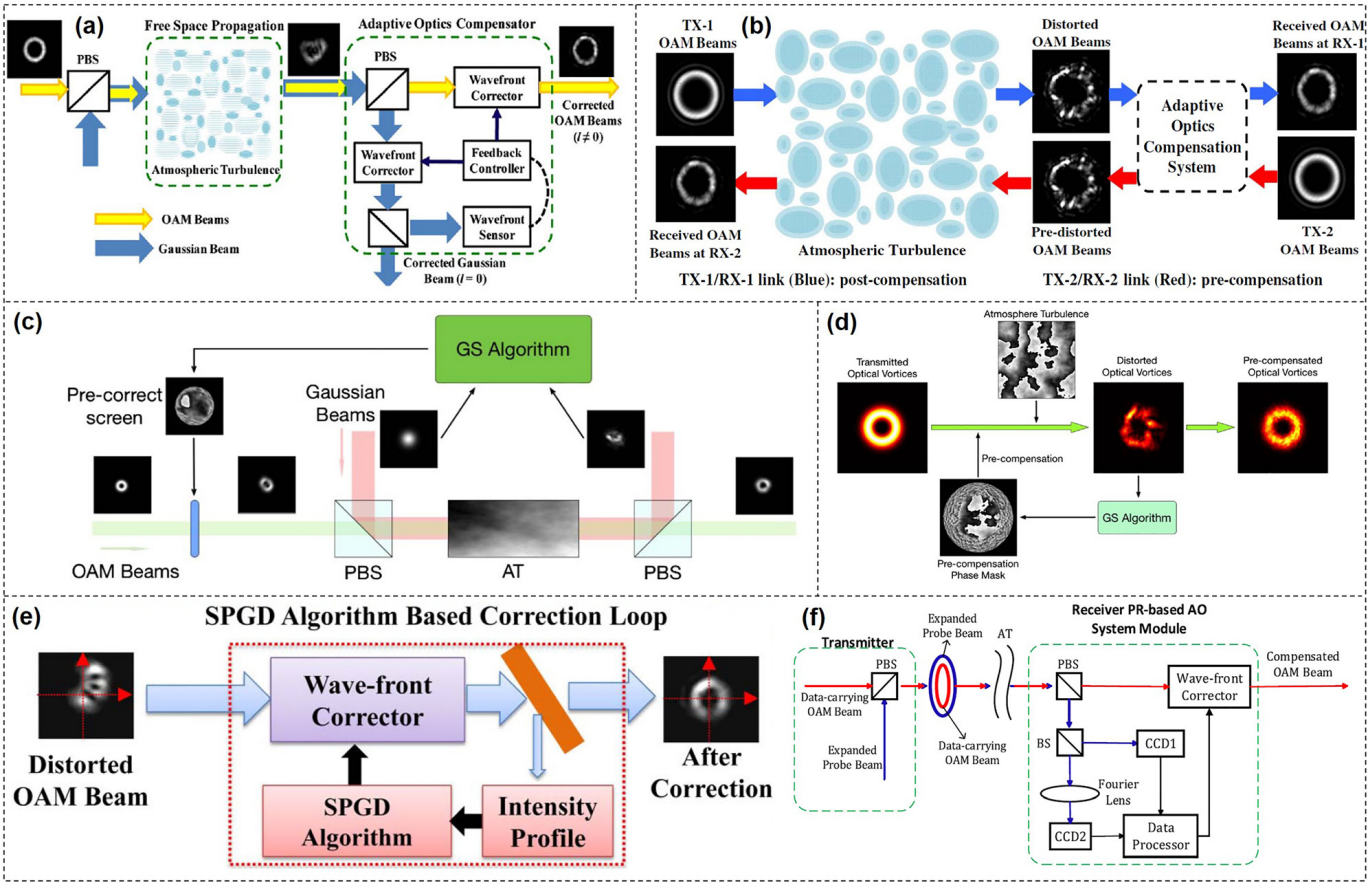
Turbulence compensation based on adaptive optics. (a) Scheme for turbulence compensation of multiple OAM beams using a Gaussian probe beam for wavefront sensing [110]. (b) Concept of simultaneous pre- and post-compensation of multiplexed OAM beams, using a single adaptive optics system [111]. (c) Scheme for the pre-turbulence compensation based on a probe and the GS algorithm [112]. (d) Concept of the GS-based non-probe pre-compensation of distorted OAM beams [113]. (e) Concept of the GS-based non-probe pre-compensation of distorted OAM beams [114]. (f) Concept of OAM communications with a hybrid input–output algorithm based adaptive optics system [115]. Reprint permission obtained from [110–115].

In general, an adaptive optics compensation system is a closed-loop configuration and consists of three steps iteratively: (i) sensing wavefront of the distorted beams, (ii) generating correction patterns according to the measured distorted phase distributions, (iii) applying correction patterns onto turbulence-distorted beams [109]. In this way, a wavefront sensor (WFS) is used to measure the wavefront of input beams. SLMs, deformable mirrors and digital micromirror devices (DMD) can be employed for wavefront correction. However, it is challenging to directly measure an OAM beam’s wavefront using the conventional WFS due to the doughnut shape of OAM beams. Thus, a separate Gaussian probe beam for phase distortion sensing was proposed to overcome these problems in adaptive optics systems. As shown in Figure 15a, a Gaussian probe beam coaxially propagated with OAM beams through atmospheric turbulence which meant that both the Gaussian beam and the OAM beams went through the same distortion [110]. At the receiver, the Gaussian beam was filtered out and sent to a WFS for wavefront measurements. The correction patterns, which were retrieved from the results, could be sent to a wavefront corrector to compensate all distorted OAM beams. In addition, considering the reciprocity of atmosphere turbulence, the beams propagating in opposite directions would experience similar turbulence distortions. Thus, an adaptive optics system could pre- and post-compensate distorted OAM beams in a bidirectional free-space optical communication link, as illustrated in Figure 15b [111].

In addition to WFS-based turbulence sensing, by using phase retrieval algorithm, such as Gerchberg–Saxton (GS) algorithm and stochastic-parallel-gradient-descent (SPGD) algorithm, the distorted wavefront could be directly retrieved from measured intensity profiles. The GS algorithm is a well-known iterative algorithm that can be used to retrieve the phase of a pair of light distributions related to a propagating function by measuring the intensities at the receiver. Figure 15c displayed a typical GS-based adaptive optics system for pre-turbulence compensation of distorted OAM beams [112]. The Gaussian probe beam was combined with OAM beams with different polarizations. Similarly, the correction patterns could be retrieved from intensity profiles of the Gaussian beam with the assist of the GS algorithm. In the above configuration, a probe Gaussian beam was still necessary which could increase the cost and complexity. In fact, the correction patterns could also be directly assessed from distorted intensities of the OAM beams by using GS algorithm. Figure 15d displayed the concept of non-probe GS-based pre-compensation of distorted OAM beams [113]. This algorithm took into consideration of both the doughnut intensity shape and the helical phase structure. The experiment results indicated that this scheme also had a favorable compensation performance. Moreover, phase correction patterns could also be generated by analyzing the measured intensity pattern together with a Zernike-polynomials based SPGD algorithm [114], as illustrated in Figure 15e. The correction patterns were approximated by a linear combination of orthogonal Zernike polynomials, and the Zernike polynomial coefficients were obtained by monitoring the intensity profile of the distorted OAM beam through an iteration-based feedback loop. The experiment results showed that the patterns derived from this method could simultaneously correct multiple OAM beams propagating through the same turbulence, and the crosstalk among these modes was reduced by more than 5 dB. In addition, the hybrid input–output algorithm (HIOA) manipulated not only the phases but also the amplitudes during the iteration. Figure 15f proposed an adaptive optics system scheme with HIOA to compensate for the distorted OAM beams [115]. The HIOA is regarded as a solution to the problem of the slow convergence of the phase retrieval algorithm.

### 7.2 Compensation based on digital signal processing

**Figure 16:**
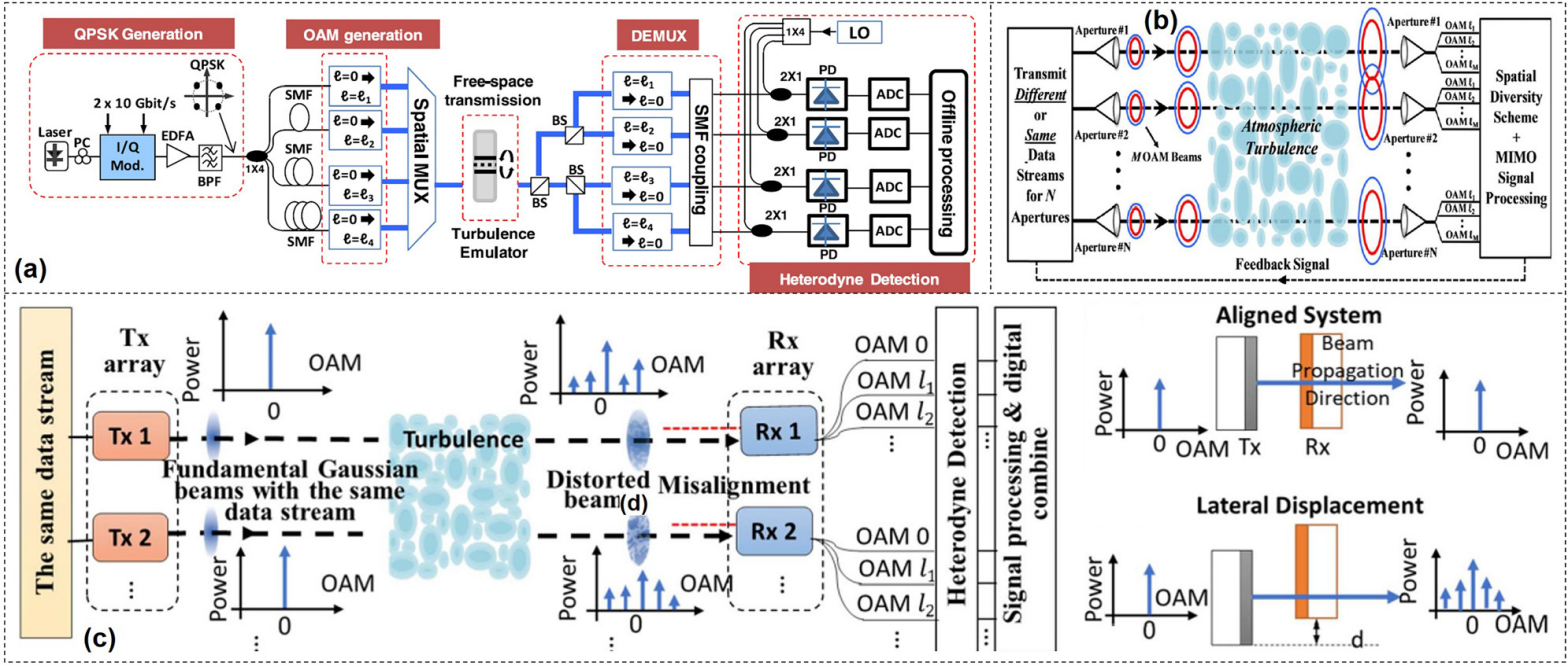
Turbulence compensation based on digital signal processing. (a) Block diagram for the demonstration of MIMO-based turbulence mitigation [116]. (b) Concept of spatial diversity assisted MIMO equalization for turbulence mitigation [118]. (c) Concept of the free-space optical communication link combining aperture diversity and the multimode receiver [119]. Reprint permission obtained from [116, 118, 119].

Digital signal processor (DSP) algorithms in the receiver can also be used to mitigate turbulence effects on OAM communication systems. With the assist of the electrical device, the complexity of the optical subsystem could be reduced, which makes the system more compact, faster and robust. Multi-channel adaptive multiple-input multiple-output (MIMO) equalizer was implemented in the receiver to reduce the crosstalk effects caused by turbulence as shown in Figure 16a [116]. After demultiplexing, each channel was combined with a local oscillator and detected by a photodiode. Due to the turbulence induced crosstalk, each channel might also have leaked power from the other channels. After optical-to-electrical conversion, the four signals from the four photodetectors (PDs) were simultaneously sampled by a four-channel real-time scope for the following offline DSP. It should be noted that, the above MIMO DSP could just work well for relative weak atmospheric turbulence. When data-carrying OAM beams propagated through strong turbulence, the crosstalk among OAM channels might exceed a certain threshold or one of the channels was barely detectable in which case MIMO would not help to improve the system performance [117]. To overcome this limit, a modified approach was demonstrated in Figure 16b, by exploiting the usage of spatial diversity combined with MIMO equalization [118]. The link included *N* transmitter/receiver aperture pairs arranged in a linear uniform structure. Each of the transmitter apertures transmitted *M* multiplexed OAM beams, having *N* × *M* OAM data channels in total. The OAM beams from different apertures overlapped spatially at the receiver aperture due to the beam divergence, which could be used by a specific spatial diversity scheme. Furthermore, aperture diversity combined with multiple-mode receivers and MIMO DSP was demonstrated to enhance the tolerance to atmospheric turbulence and spatial misalignment as shown in Figure 16c [119]. In this scheme, the robustness of link was enhanced by using multiple fundamental Gaussian beams with the same data between the transmitter and receiver aperture pairs. For each aperture pair, the turbulence effects as well as the aperture misalignment (e.g., lateral displacement) would induce power loss on the fundamental Gaussian mode and power coupling to its neighboring modes. At the receiver, the data over multiple modes and multiple receiver apertures were digitally combined using MIMO DSP.

### 7.3 Compensation based on auto-alignment system

Besides adaptive optics and digital signal processing to correct wavefront distortions caused by atmospheric turbulence [111, 120], the auto-alignment system is also an alternative method to correct the beam displacement and optimize the coupling efficiency to against the turbulence. We proposed and experimentally demonstrated a free-space adaptive optics communication link against atmospheric turbulence and device vibration by introducing Zernike Polynomials based SPGD algorithm and a fast auto-alignment system [25]. The transmission performance using 16-QAM signals in the case of adaptive system assistance was studied, showing the power penalty improvement of 8 dB with the adaptive system.

**Figure 17:**
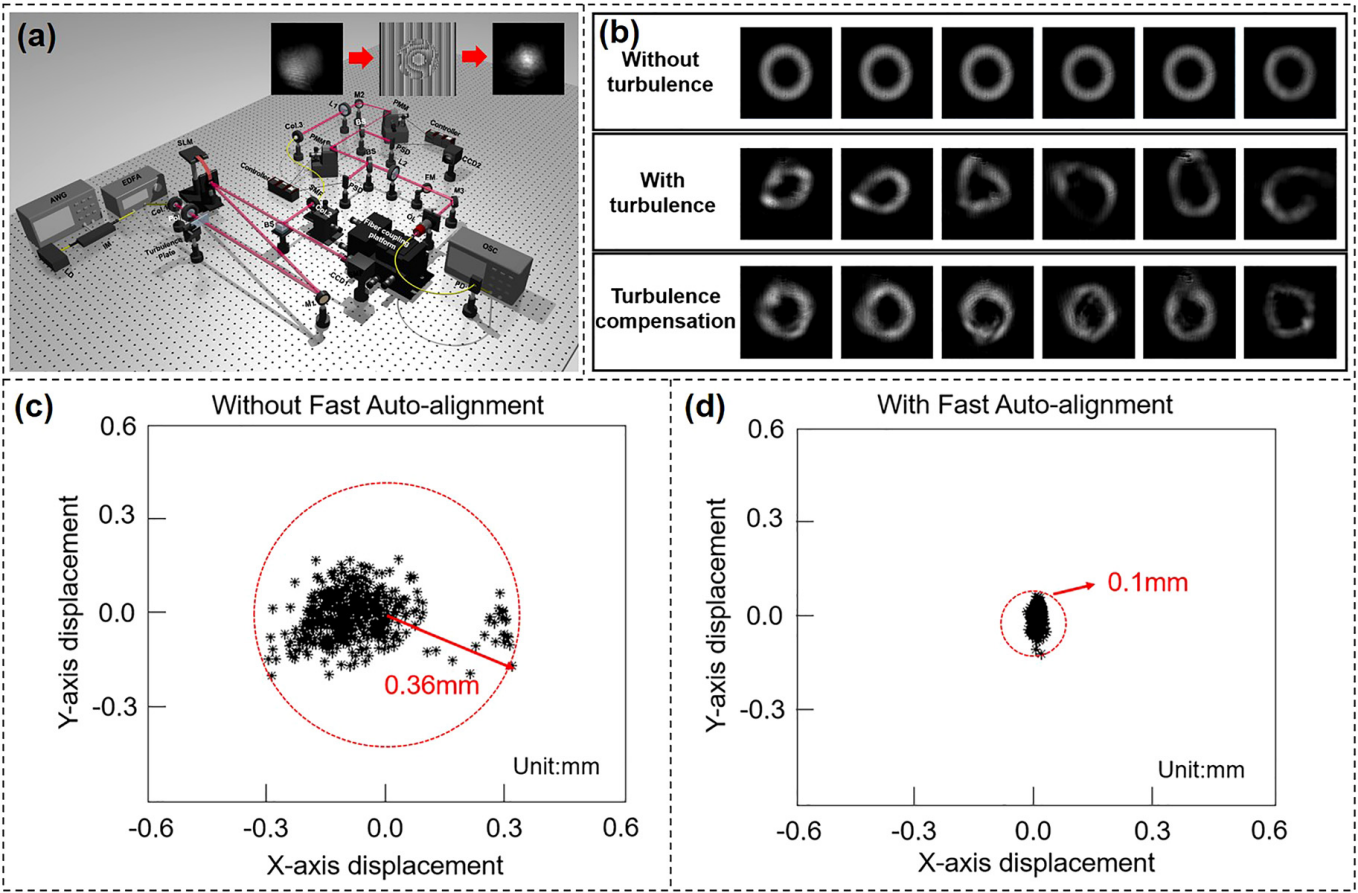
Turbulence compensation based on auto-alignment system. (a) Experimental setup of free-space adaptive optics communication link against atmospheric turbulence and device vibration. PMM: piezo mirror mount; SMF: single-mode fiber; PSD: position sensing detector; FM: flip mirror; OL: objective lens; OSC: oscilloscope. (b) Intensity profiles of the OAM beam (*l* = +3) recorded by 1550 nm camera under three different conditions: without turbulence, with turbulence and without compensation, with turbulence and with compensation. (c) Measured beam displacements without fast auto-alignment system. (d) Measured beam displacements with fast auto-alignment system [25]. Reprint permission obtained from [25].

Figure 17a showed the experimental setup of the free-space adaptive optics communication link assisted by a fast auto-alignment system. A 1550 nm light beam was modulated by an intensity modulator to carry 10-Gbaud 16-QAM signal generated by an arbitrary waveform generator (AWG). The atmospheric turbulence was introduced by a turbulence plate. After passing through the turbulence plate, the amplified and collimated light was injected onto an SLM loaded with a phase hologram to compensate the turbulence. Then the light beam was split into two paths by a beam splitter. One of the beams was captured by the CCD1, which was used to monitor the intensity profiles of light through turbulence in order to determine the hologram loaded onto the SLM. The other beam would pass through the fast auto-alignment system which comprised two alignment stages. Each of them was formed by one quadrant detector, one position sensing detector (PSD) auto aligner, two piezo controllers, one BS, and one piezo mirror mount (PMM). The displacement of an incident beam away from the center was sent from the quadrant detector to PSD auto aligner to calculate the drive voltage given by two piezo controllers. Figure 17b displayed intensity profiles of the OAM beam (*l* = +3) under three different conditions with different turbulence conditions: without turbulence (the first row), with turbulence and without compensation (the second row), with turbulence and with compensation (the third row). Compensation for turbulence made the intensity profiles of OAM beams more regular and certainly, closer to the center of the picture.

To emulate the vibration conditions, mirror M2 was placed on a motor whose vibration frequency was measured to be about 196 Hz. When the light beam was incident on the oscillatory mirror, the light would be reflected on a random position and orientation. By switching on the auto-alignment system, the beam displacement was measured by the quadrant position sensor which was sent to the DSP inside the PSD. Then the piezo driver operated together with the PSD auto aligner to get high precision closed loop operation for the alignment by using the complete range of feedback. As shown in Figure 17c, in the absence of an auto-alignment system, the beam exhibited a distribution range of 0.36 mm under the influence of motor vibration, which would greatly reduce the power at the receiving end resulting in poor performance of the communication link. By introducing the fast auto-alignment system, the beam vibration range was controlled to be 0.1 mm as shown in Figure 17d, reducing the spatial range of beam vibration caused by device vibration by 72.22%.

## 8 Structured light communications beyond OAM

### 8.1 Communications using Bessel beams

Bessel beam can reconstruct its electric field after passing through an obstruction which is beneficial for free-space optical communications. It might achieve superior performance when there exists an obstruction in the free-space link. We experimentally demonstrated 10 Bessel beams multicasting from a Gaussian beam with a single optimized designed multi-Bessel phase pattern [121]. We also demonstrated four Bessel beams multicasting carrying 20 Gbit/s QPSK signal. Moreover, by setting an obstruction in the free-space link, the multicasted four Bessel beams showed relatively low crosstalk (<−10 dB) from their neighboring Bessel beams.

**Figure 18:**
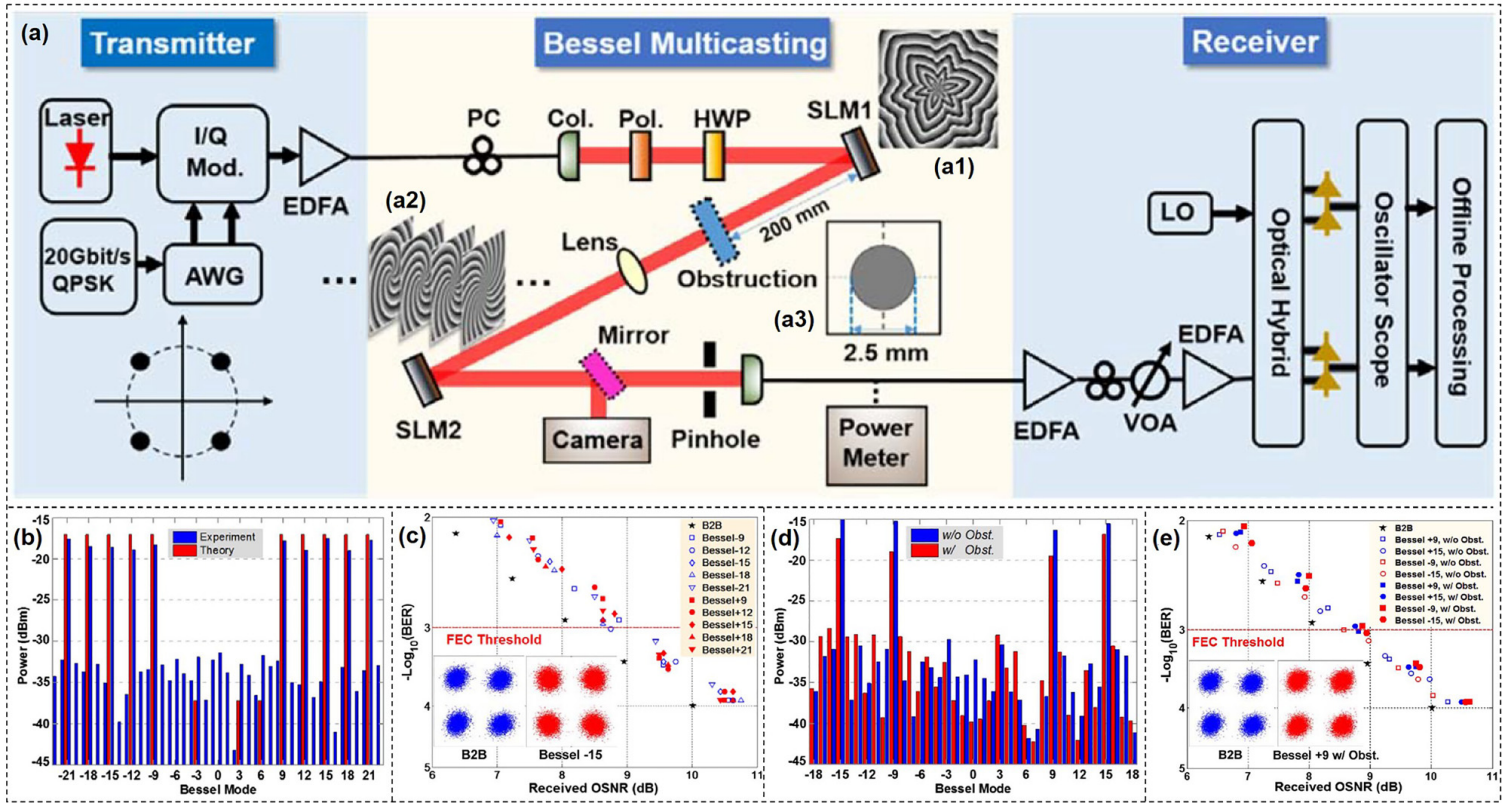
*N*-fold Bessel beams multicasting. (a) Experimental setup of *N*-fold Bessel beams multicasting. (a1) Phase pattern for generating multiple Bessel beams. (a2) Phase pattern for back converting a Bessel beam to a Gaussian-like beam. (a3) Obstruction pattern. (b) Measured and theoretical power distribution for 10 Bessel beams multicasting (*l* = ±9, ±12, ±15, ±18, and ±21). (c) Measured BER performance of all channels (*l* = ±9, ±12, ±15, ±18, ±21) for 10 Bessel beams multicasting. (d) Measured and theoretical power distribution for four Bessel beams multicasting (*l* = ±9 and ± 15). (e) Measured BER performance for four Bessel beams multicasting with and without obstruction [121]. Reprint permission obtained from [121].

Figure 18a displayed the experimental setup of *N*-fold Bessel beams multicasting. A Gaussian beam carrying 20-Gbit/s QPSK signal at a wavelength of 1550 nm was generated at the transmitter. SLM1 loaded with a multi-Bessel hologram was used to realize Bessel beam multicasting as shown in Figure 18a1. After propagating in free space, the duplicated Bessel beams were back converted to a Gaussian-like beam by SLM2, followed by coherent detection which was used to analyze the QPSK signal to evaluate the performance of *N*-fold data-carrying Bessel beam multicasting.

We first evaluated the performance of 10 Bessel beams multicasting by measuring the power distribution over all Bessel channels as shown in Figure 18b. The observed extinction ratio for all 10 OAM-carrying Bessel channels was large than 15 dB which was in agreement with the theory. The BER performance of 10 Bessel beams multicasting was shown in Figure 18c with observed OSNR penalties of about 0.7 dB at a BER of 1e-3 for the multicasting Bessel channels. Moreover, we also measured Bessel spectra for four Bessel beams multicasting with and without obstruction as displayed in Figure 18d. The received power with obstruction was about 3 dB lower than that without obstruction as the beam was partly blocked by the obstruction. The BER performance of four Bessel beams multicasting with and without obstruction was shown in Figure 18e. Compared to the unobstructed channels, the ones with obstruction had about 0.8-dB OSNR penalties, which showed almost the same performance as the unobstructed channels.

**Figure 19:**
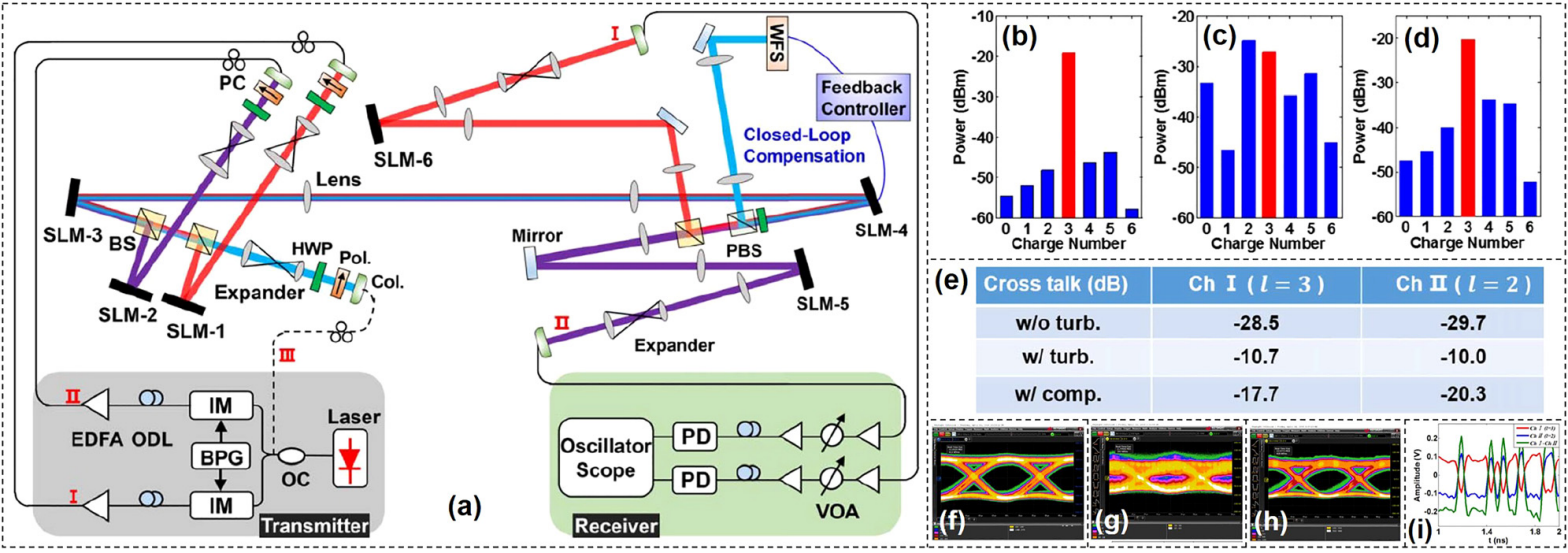
High-speed Bessel beams encoding/decoding. (a) Experiment setup of high-speed encoding/decoding of Bessel beams through turbulence assisted by adaptive compensation. OC, optical coupler; BPG, bit-pattern generator; IM, intensity modulator; ODL, optical delay line; EDFA, erbium-doped fiber amplifier; PC, polarization controller; SLM, spatial light modulator; Col., collimator; Pol., polarizer; HWP, half-wave plate; BS, beam splitter; PBS, polarization beam splitter; WFS, wavefront sensor; VOA, variable optical attenuator; PD, photodetector. Measured power distributions of various channels with only path Ⅰ on (b) without turbulence, (c) before and (d) after compensation with turbulence. (e) Measured crosstalks for channels Ⅰ and Ⅱ in the cases without turbulence (w/o turb.), with turbulence (w/turb.), and with compensation (w/comp.) of 20-Gbit/s Bessel beam encoding/decoding link. Eye diagrams of Ch Ⅰ for (f) back-to-back, Bessel beam decoding (g) before and (h) after compensation. (i) Temporal waveforms of two channels (Ch Ⅰ, Ch Ⅱ) and their subtraction (Ch Ⅰ–Ch Ⅱ) after turbulence compensation [71]. Reprint permission obtained from [71].

Compared to the OAM beam, Bessel beam can effectively decrease signal distortion when there is an obstruction in the propagation path in the free-space link due to the characteristics of propagation invariance or diffraction free. We experimentally demonstrated an adaptive optics compensation technique for a turbulence-distorted 20-Gbit/s Bessel beam encoding/decoding link. The experimental setup was shown in Figure 19a [71]. Two light beams at a wavelength of 1550 nm were modulated by two intensity modulators which were driven by two opposite OOK data sequences from a bit-pattern generator. The two modulated Gaussian beams launched into two SLMs which were loaded with different holograms to generate different Bessel beams, i.e., *l* = 3 (path Ⅰ/Ch Ⅰ) and *l* = 2 (path Ⅱ/Ch Ⅱ). The two Bessel beams were combined together by a beam splitter to get a high-speed time-varying Bessel beam sequence. Another Gaussian beam was introduced by an additional beam splitter to sense the phase distortion of the turbulence. SLM3 was used to emulate the turbulence by loading a pseudorandom turbulence hologram, while SLM4 worked as a wavefront corrector to form a compensation close-loop with a WFS. A feedback controller was used to dynamically feed SLM4 with the appropriate correction masks by measuring the phase difference between the target phase and actual phase of the probe Gaussian beam. The compensated Bessel beam sequence was divided into two paths for demodulation and detection.

We first studied the performance of the turbulence compensation. Figure 19b–d illustrated the power distribution without turbulence, before and after compensation with turbulence, respectively. It was shown that the compensation close loop had favorable performance to decrease the severe interchannel crosstalk introduced by turbulence. Furthermore, we measured crosstalks both for Ch I (*l* = 3) and Ch II (*l* = 2) in the cases without turbulence, with and without compensation as shown in Figure 19e. Finally, we measured the BER performance of the 20-Gbit/s high-speed Bessel beam encoding/decoding link with random moderate turbulence. The eye diagrams of Ch I for back-to-back, Bessel beam decoding before turbulence compensation, and Bessel beam decoding after turbulence compensation were depicted in Figure 19f–h, respectively. Figure 19i showed the recorded temporal waveforms of two channels (Ch I, Ch II) and their subtraction (Ch I–Ch II) after turbulence compensation. The obtained results indicated successful implementation of a 20-Gbit/s high-speed Bessel beam encoding/decoding link with adaptive turbulence compensation.

### 8.2 Communications using Airy beams

Bendable light beams are another interesting class of electromagnetic waves associated with a localized intensity maximum that propagates along a curved trajectory. Airy beam is one type of nondiffracting beams, which can maintain its wavefront during transmission just like the Bessel beam. By exploiting curved light beams instead of traditional Gaussian beam for line-of-sight optical communications, we proposed and demonstrated the viability of free-space data-carrying bendable light communications along arbitrary trajectories with multiple functionalities [122].

**Figure 20:**
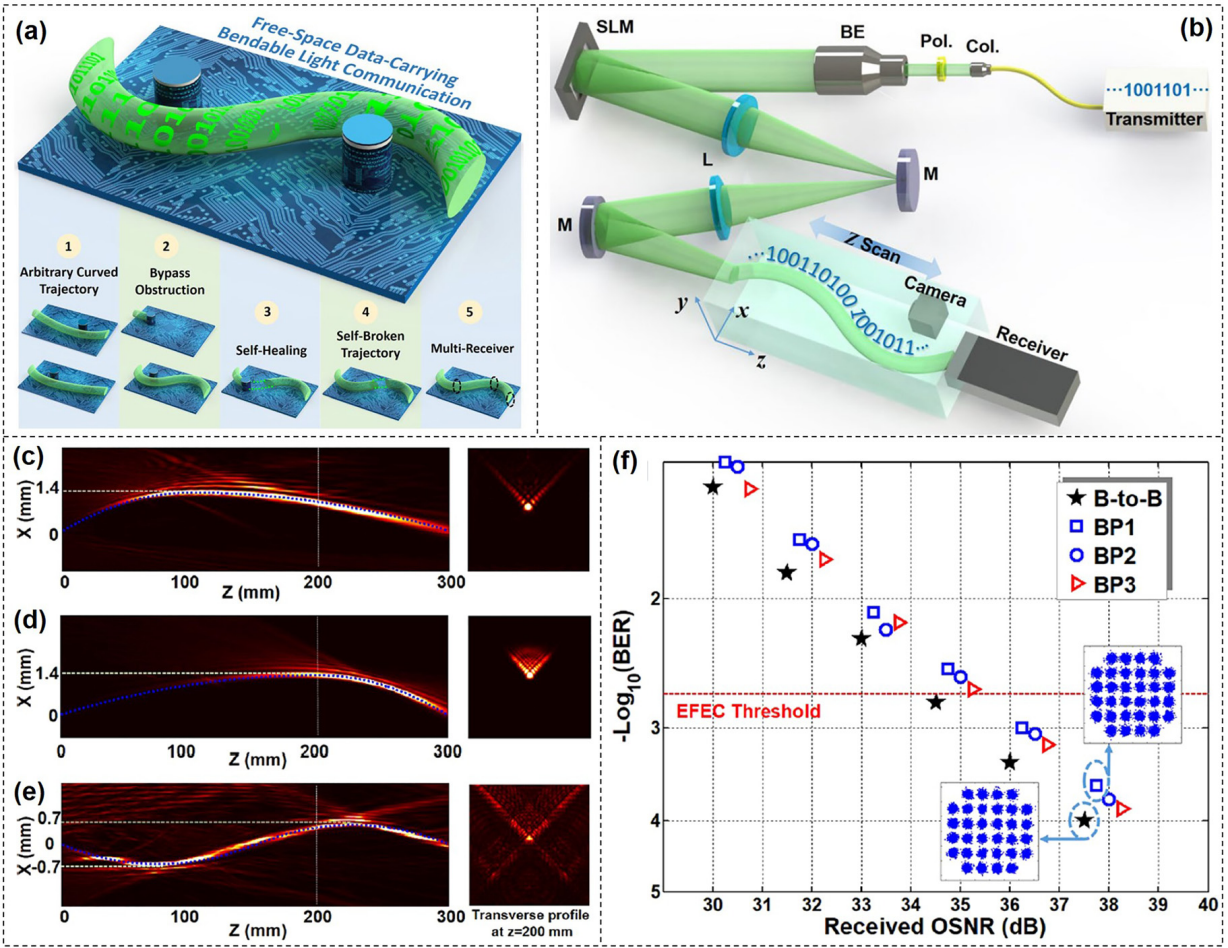
Free-space data-carrying bendable light communications. (a) Concept and principle of free-space data-carrying bendable light communications. (b) Experimental configuration of free-space bendable light communications. Col.: collimator; Pol.: polarizer; BE: beam expander; SLM: spatial light modulator; M: mirror; L: lens. (c–f) Experimental results of free-space bendable light communications along arbitrary trajectories. (c–e) Measured intensity distributions of three different bendable light beams at *x*–*z* plane (the blue dashed line was the preset trajectory) and corresponding transverse intensity profiles at *z* = 200 mm. (f) Measured BER performance of the three different data-carrying bendable light beams. Insets showed constellations of 32-QAM DMT signals [122]. Reprint permission obtained from [122].

The concept and principle of free-space bendable light communications were illustrated in Figure 20a. Firstly, with the help of optical light caustic method, we could get arbitrarily curved light paths by designing the specific phase hologram. Then, the generated bendable light could bypass existed obstructions as designed light path. After passing through the obstruction, the bendable light could recover its wavefront which made the communication system more robust. In addition, one could even construct a self-broken trajectory curved light beam, which could avoid eavesdroppers. Moreover, owing to the self-healing property, the curved light could transmit the information to multi-users along the curved light path. Consequently, by employing bendable light, the free-space communication system would become more multifunctional, more flexible and more robust. The experimental setup of free-space bendable light communications was shown in Figure 20b. A Gaussian beam carrying 39.06-Gbit/s 32-QAM discrete multi-tone (DMT) signal at a wavelength of 1550 nm was generated at the transmitter. The light beam was expanded to illuminate the full SLM loaded with the desired phase pattern by optical light caustic method for bendable light beam generation. A 4f imaging system was introduced to record the full propagating trajectory. By placing a camera after 4f system, the propagation dynamics of the bendable light was recorded by moving along a motorized linear translation stage.

Three bendable light beams with different curved trajectories were successfully generated with the intensity distributions depicted in Figure 20c–e. The propagating distances of the curved light beams were all 300 mm along the *z* direction. Figure 20c and d depicted curved light beams along parabolic trajectories with the bending offset equaling to 1.4 mm. In addition, we also generated S-shaped curved light beam as displayed in Figure 20f. The bending offset of two peaks were both 0.7 mm. One could clearly find that the measured bendable light beams were in good agreement with the predesigned trajectories as marked by blue dashed lines shown in Figure 20c–e. Furthermore, BER performance as a function of the received OSNR for the three bendable light beams was plotted in Figure 20f. The observed OSNR penalties at a BER of 2e−3 (EFEC threshold) for the three bendable light beams were ∼0.9 dB.

**Figure 21:**
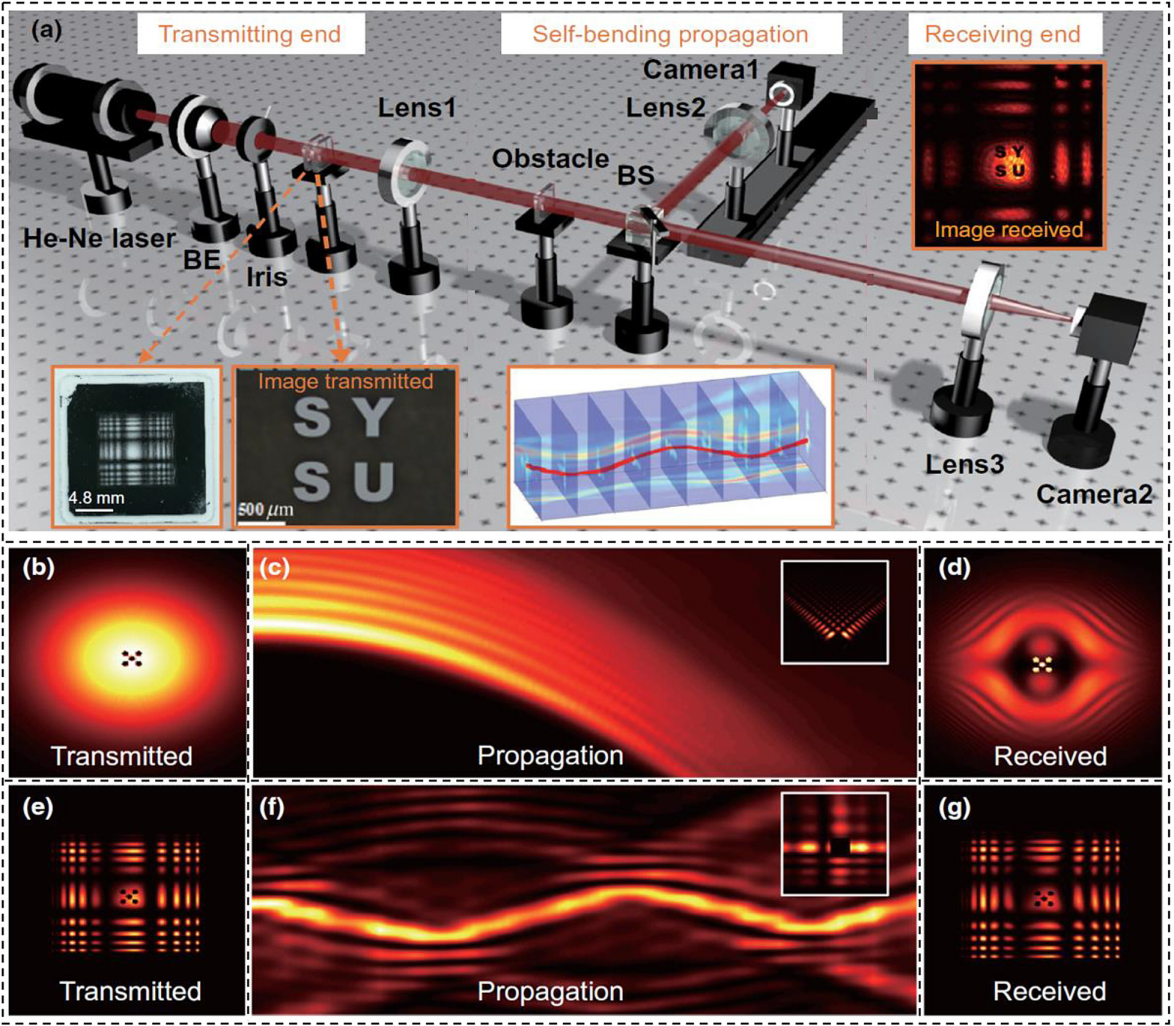
Image-signal transmission based on a nonconvex helical beam compared with the commonly used Airy beam. (a) Experimental implementation of optical image transmission based on a nonconvex accelerating beam. (b) The Fourier-space angular spectrum of the Airy beam with an encoded image. (c) Two-dimensional projection of the propagation dynamics of the Airy beam and (d) the retrieved angular spectrum of the Airy beam after blocking of its main lobe in the initial plane [as shown in the inset in (c)]. (e)–(g) Similar images as for (b)–(d) but for the case of a helical beam. BE, beam expander; BS, beam splitter [123]. Reprint permission obtained from [123].

For Airy beams, the spatial position maps to a specific spatial frequency along the main lobe. Thus, the angular spectrum would be destroyed by an obstruction in a certain plane during transmission correspondingly, which would lead to the loss of the transmitted signal. To solve this problem, the nonconvex type of accelerating beams, e.g., sinusoidal or sinusoidal-based beams with multiple endings, were introduced. For these kinds of beams, one spatial frequency corresponds to more than one position of the main lobe. As a consequence, the affection of the obstruction along the transmission would be weakened. Figure 21a showed the experimental implementation of optical image transmission based on a nonconvex accelerating beam [123]. The setup was divided into three parts: transmitting end, self-bending propagation, and receiving end. Figure 21b–g demonstrated the image transmission based on convex and nonconvex accelerating beams for comparison. Figure 21b and e were the same image represented by a pattern of five dots encoded into the central angular spectra of two kinds of beams respectively. As shown in Figure 21d, the image signal was destroyed or even appeared with contrast reversal when the main lobe of the beam was blocked in a certain plane along propagation. However, the transmitting image could still be retrieved clearly as shown in Figure 21g, and no contrast reversal appeared as there were always other positions along the main lobe mapping to a low-frequency component.

### 8.3 Communications using vector beams

Polarization is a distinct characteristic and part of the intrinsic nature of light. Particularly, vector beams with space-variant states of polarization (SoPs) have attracted increasing interest compared to homogeneously polarized beams recently [124], [125], [126].

**Figure 22:**
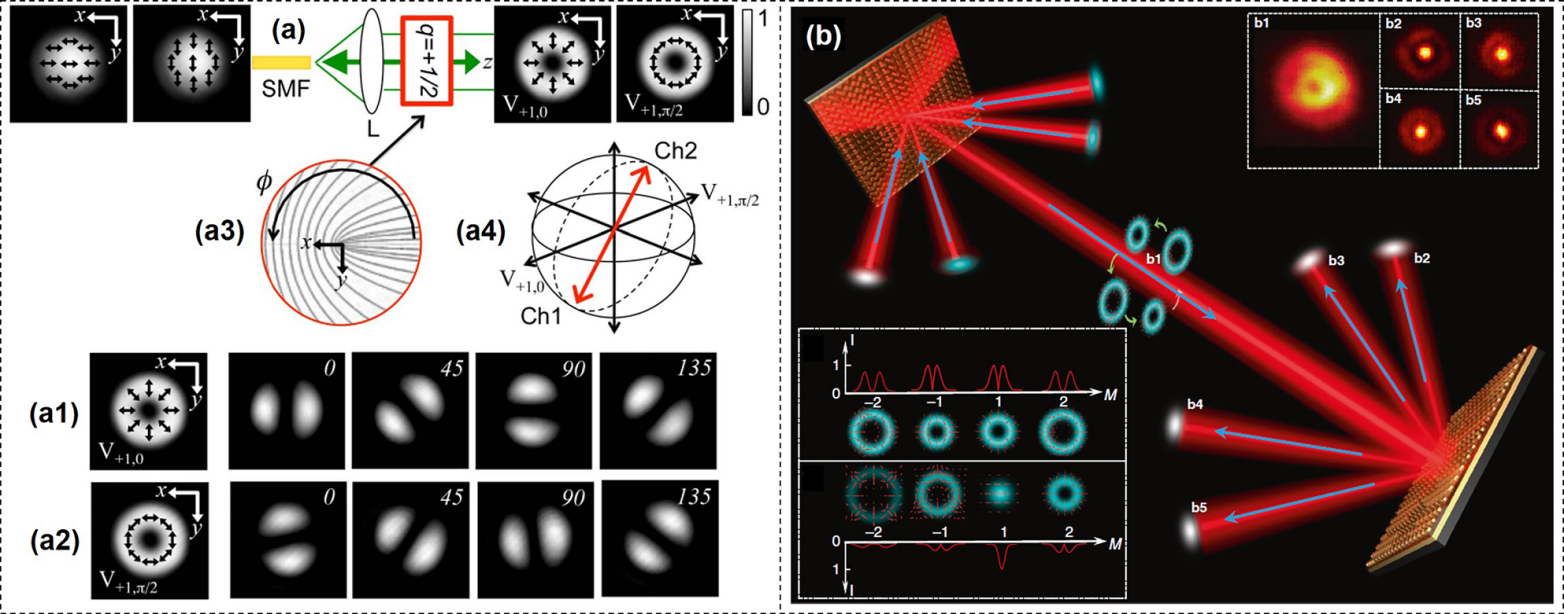
Vector beams multiplexing communications. (a) Transformation of the state of polarization of the fundamental mode of an SMF into a linear combination of vector modes using a liquid crystal q-plate as described in the text: *q* = 1/2 plate [127]. (b) Schematic of vector beams multiplexing/demultiplexing by using metasurface-based Dammann optical vector gratings [128]. Reprint permission obtained from [127, 128].

In 2014, four vector modes were used to increase the transmission data rate in which a liquid crystal q-plate was introduced as the mode (de)multiplexer for vector beams as shown in Figure 22a [127]. A q-plate comprises a thin layer of patterned liquid crystal molecules in-between two thin glass plates. Four vector beams each carrying a 20-Gbit/s QPSK signal (aggregate 80 Gbit/s) on a single wavelength channel (λ∼1550 nm) were transmitted ∼1 m over the lab table, with mode crosstalk lower than −16.4 dB. BER for all vector modes was measured at the 7% FEC threshold with power penalties lower than 3.41 dB. As mentioned before, a large variety of devices have been proposed to generate structured light beams including vector beams, such as q-plate, SLM and so on. The spin-dependent Pancharatnam–Berry (P–B) phase devices have been explored to separate vector beams. The mechanism is to independently control the wavefront of the respective left- and right-handed circularly polarized (LHCP/RHCP) components as vector beams can be theoretically decomposed into two vortex beams with opposite-handed circular polarization and conjugate topological charges. As shown in Figure 22b, off-axis control of polarization was realized with metal–dielectric–metal metasurface by combining the P–B phase with the propagation phase [128]. The fabricated (de)multiplexers were broadband ranging from 1310 to 1625 nm. By using this device, a four-channel vector beams (m = ±1, ±2) multiplexing communication combining WDM and PDM was demonstrated, with a transmission rate of 1.56 Tbit/s and a BER of 10^−6^ at the received power of −21.6 dBm.

Besides vector beams multiplexing, we demonstrated a visible-light hexadecimal vector beam array modulation for parallel communication link, carrying 4 × 6/6 × 8/12 × 16 hexadecimal numbers with error-free data stream transmission [129].

**Figure 23:**
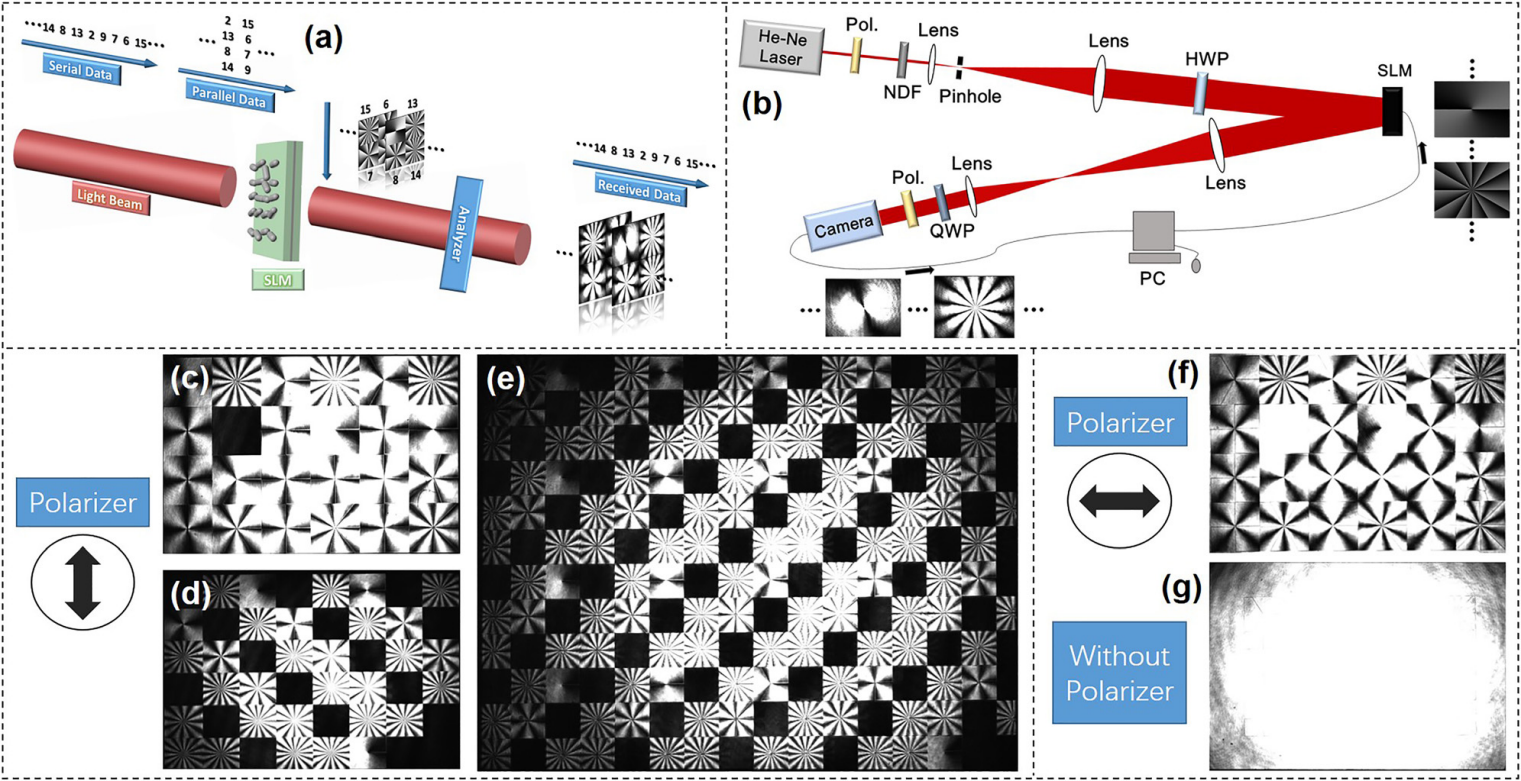
Vector beam array modulation communications. (a) Concept and principle of hexadecimal modulation using vector beam array in a visible-light parallel communication link. (b) Schematic of the experimental setup for hexadecimal modulation using vector beam array in a visible-light parallel communication link. Pol.: polarizer; NDF: neutral density filter; HWP: half-wave plate; SLM: spatial light modulation; QWP: quarter-wave plate. Intensity distribution of hexadecimal vector beam arrays analyzed by *y*-direction polarizer with the elements of (c) 4 × 6, (d) 6 × 8 and (e) 12 × 16 respectively. Power distribution of 4 × 6 array analyzed by *x*-direction (f) with polarizer and (g) without polarizer [129]. Reprint permission obtained from [129].

The concept and principle of hexadecimal modulation using vector beam array in a visible-light parallel communication link were shown in Figure 23a. The input Random hexadecimal serial data were converted to parallel data according the capacity of the array. As shown in Figure 23a, the serial data were converted to an *m* × 4 matrix as the vector beam array carrying 2 × 2 hexadecimal numbers. Every row of the matrix corresponded to a pattern loaded onto the SLM, which was continuously switched according to the parallel data stream to generate vector beam array. By passing through the analyzer formed by a quarter-wave plate (QWP) and polarizer, a petal-like intensity distribution could be observed on the CCD. The parallel transmission data information could be recovered to serial data by the mapping relationship with the number of the petal. Figure 23b displayed the setup of the vector beam array modulation link. The light beam from He–Ne laser was expanded by two lenses to get as large as possible vector beam array, which meant a larger data capacity. The half-wave plate (HWP) was used to rotate the polarization to 45° corresponding to the modulation polarization of SLM, resulting in half of the beam modulation. Then the light beam was followed by another two lenses to reduce the beam for detection by the CCD. The QWP (45° rotated) and polarizer worked as the analyzer to decode the vector beam array.

Figure 23c–e showed the intensity distribution of hexadecimal vector beam arrays with 4 × 6, 6 × 8 and 12 × 16 elements. In principle, the information capacity increased with the number of the elements. However, considering the spatial inhomogeneous Gaussian power distribution, elements located in the margin of array had lower contrast between the bright petals and the dark ones which increased the complexity of the decoding process. Therefore, 4 × 6 hexadecimal vector beam array was demonstrated for parallel data stream transmission, which contained 24 hexadecimal numbers, or 96 bits per state. A corresponding algorithm was used to convert the elements of the received vector beam array to parallel data which was recovered to decimal data stream later.

## 9 Towards diverse and robust communications using OAM and beyond

### 9.1 OAM communications in multiple scenes

**Figure 24:**
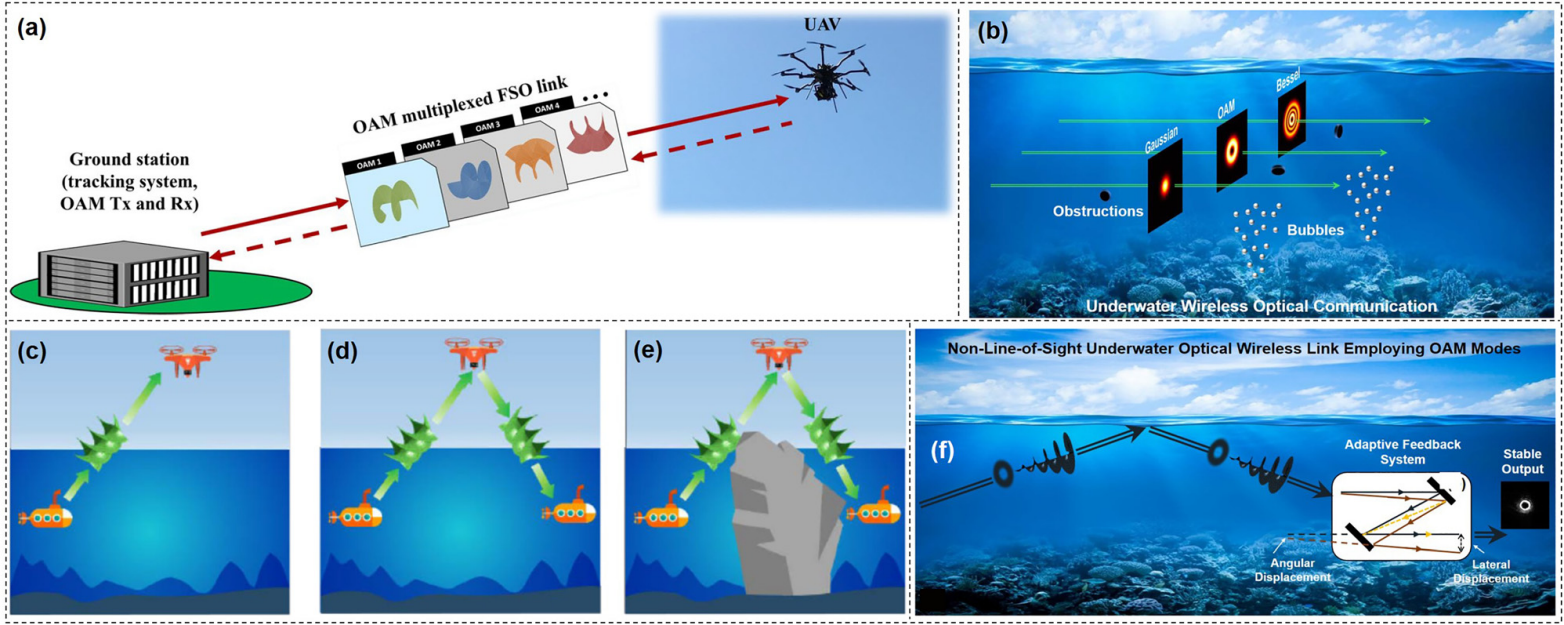
Diverse OAM communications in multiple scenes. (a) Concept of a free-space optical communication link between an unmanned-aerial-vehicle (UAV) and a ground station using OAM multiplexing [130]. (b) Concept of underwater wireless optical communications employing three different spatial modes (Gaussian, OAM, and Bessel) subjected to bubbles and obstructions [131]. (c–e) Concept and principle of collaborative communication scenarios with underwater twisted green light across the air-to-water interface [132]. (c) Communication between an air user and an underwater user. (d) Collaborative communication between an air user and two underwater users. (e) Reflection communication between an air user and two underwater users. (f) Concept and principle of non-line-of-sight underwater optical link employing OAM beams [133]. Reprint permission obtained from [130–133].

A scheme was proposed to increase the capacity of free-space data transmission on moving platforms in the real turbulence, with the potential benefit of maneuvering ability and decreasing the probability of data intercept [130]. To be specific, it demonstrated and characterized the performance of an OAM-multiplexed free-space optical communication link between a ground transmitter and a ground receiver via a moving unmanned-aerial-vehicle (UAV). This scheme extended the OAM multiplexing application field from static stations to moving devices, leaving massive space to explore [130]. In the aspect of information capacity, it achieved a total capacity of 80 Gbit/s up to 100-*m*-roundtrip link by multiplexing two OAM beams, each carrying a 40-Gbit/s QPSK signal. Figure 24a illustrated the prospective application for using OAM multiplexing in high-capacity free-space optical communications between a hovering UAV and a ground station with a tracking system as well as an OAM transmitter and receiver [130].

Ocean has been widely studied in scientific, commercial, as well as military in the history. Optical wave, especially for the blue-green region with relative low attenuation, enables higher capacity and spectrum efficiency for underwater wireless optics communication (UWOC). Beyond OAM communications in free space, the combination of free-space optical communications with other communication scenes, for example UWOC, is also desired in the communication system. Figure 24b demonstrated an UWOC link using different spatial modes (Gaussian, OAM and Bessel) subjected to bubbles and obstructions [131]. The bubbles were imitated by a commercial oxygen pump which was a dynamic interference, while the obstructions, which were a static interference, were introduced into the optical path artificially. The underwater transmission performance was evaluated by employing three spatial modes carrying 1.4 Gbaud OFDM 16-QAM signals. Figure 24c–e demonstrated an adaptive water-air-water transmission link employing OAM beams [132]. The change of water surface height caused the misalignment of light beam which would decrease the detection efficiency. By introducing a reflection element in the system, the received intensity distribution was adjusted due to the feedback. The water surface height in the tank was changed by filling water to emulate the ebb and flow of the tide in true situation. The light beam went through water-to-air interface with the help of two mirrors fixed at the bottom of the tank. The reflection element was set above the tank to steer the beam light for feedback of the intensity. After passing through the tank, the beam was reduced by two lenses to be demodulated by an SLM. The light path could be kept with the original one by adjusting the height of the feedback-assisted reflection element to ensure successful data transmission when the water surface height changed. Figure 24d presented a non-line-of-sight UWOC employing OAM by the total internal reflection on the air-water interface [133]. Natural phenomena such as the slight wind, the salinity (turbidity) and the vertical thermal gradient-induced turbulence would change the interface state and transmission link performance. Usually, a large receiver aperture was introduced to enlarge the field-of-view (FOV). As for OAM communications, large FOV was not enough for signal recovery as the demodulation of OAM needed more precise optical path alignment. An adaptive feedback system at the receiver side for OAM UWOC in such non-line-of-sight scenario to guarantee the signal quality was shown in Figure 24f.

### 9.2 Turbulence-resilient structured light communications

As mentioned before, atmospheric turbulence is an important factor affecting the performance of free-space optical communications. Apart from the common techniques introduced before to mitigate turbulence effects, there are some other approaches to improve the performance of the structured light communications in turbulence.

**Figure 25:**
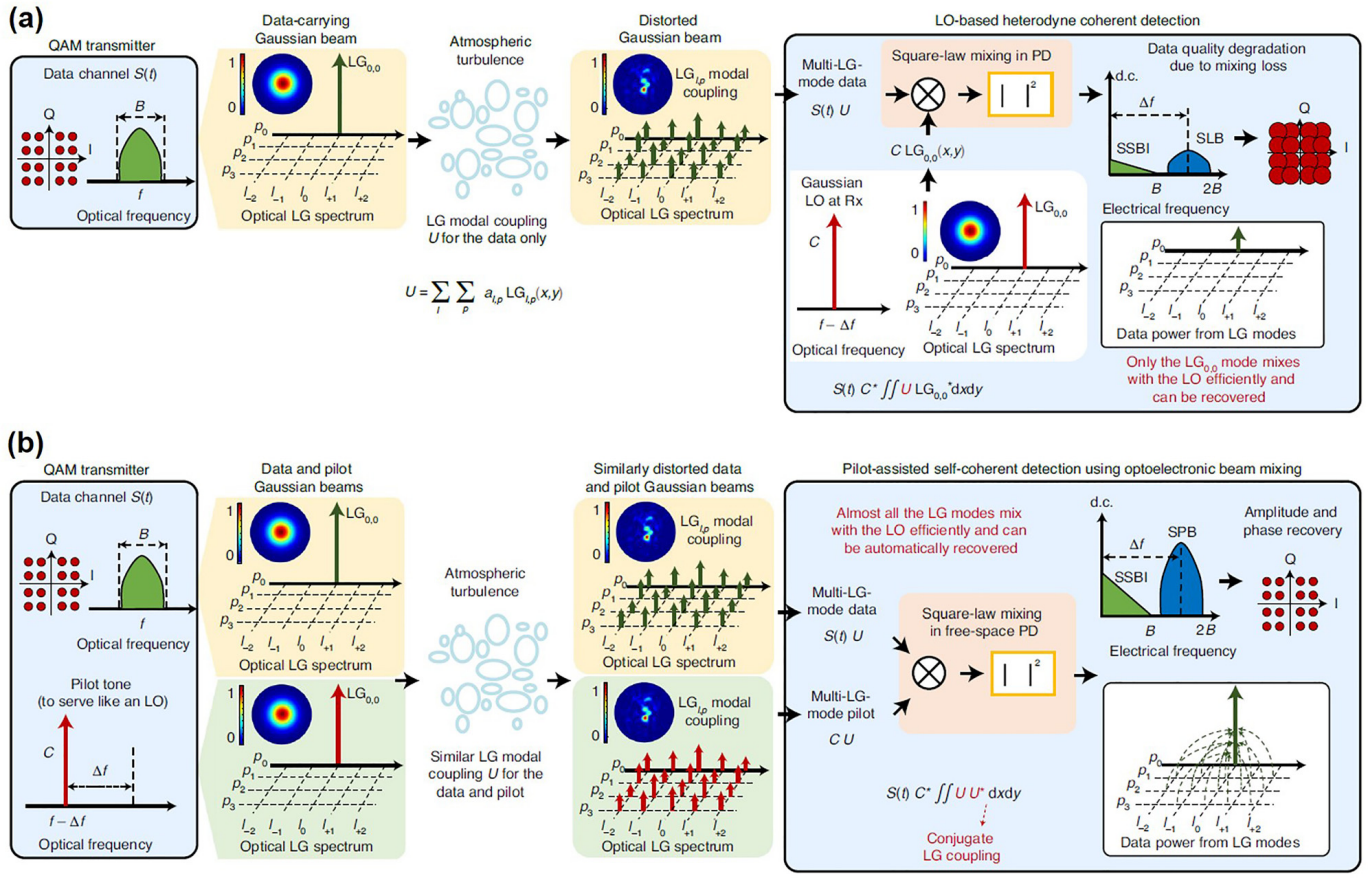
Concept of simultaneous amplitude and phase recovery of data in turbulent free-space optical communication links. (a) The performance of coherent detection could be significantly degraded by turbulence-induced LG modal-coupling effects. (b) Pilot-assisted self-coherent detection could automatically compensate for the turbulence-induced LG modal-coupling effects [134]. Reprint permission obtained from [134].

For a light beam modulated by both amplitude and phase, e.g., light beam carrying *m*-QAM signal, the data could be recovered by mixing a Gaussian local oscillator (LO) with the received Gaussian data beam. However, power coupling between transmitted Gaussian mode and higher-order modes induced by atmospheric turbulence would degrade the mixing efficiency and system performance dramatically. As shown in Figure 25a, a fundamental Gaussian beam (LG_0,0_ mode) carrying 16-QAM data was transmitted through a turbulent atmosphere. At the receiver, the light beams would contain many LG modes due to the turbulence-induced LG modal power coupling. In a LO-based heterodyne coherent detector, only the LG_0,0_ mode could be efficiently mixed with the LO and recovered, resulting in degradation of the recovered data quality. Figure 25b demonstrated a pilot-assisted self-coherent detection approach to overcome this problem [134]. Specifically, a Gaussian data beam and a frequency-offset Gaussian pilot tone beam were transmitted at the same time to experience similar turbulence and modal coupling. Subsequently, a photodetector mixed all corresponding pairs of the beams’ modes. During mixing, a conjugate of the turbulence-induced modal coupling was generated and compensated the modal coupling experienced by the data, and thus the corresponding modes of the pilot and data mixed efficiently. A 12-Gbit/s 16-QAM polarization-multiplexed free-space optical communication link was demonstrated in the experiment that was resistant to turbulence [134].

**Figure 26:**
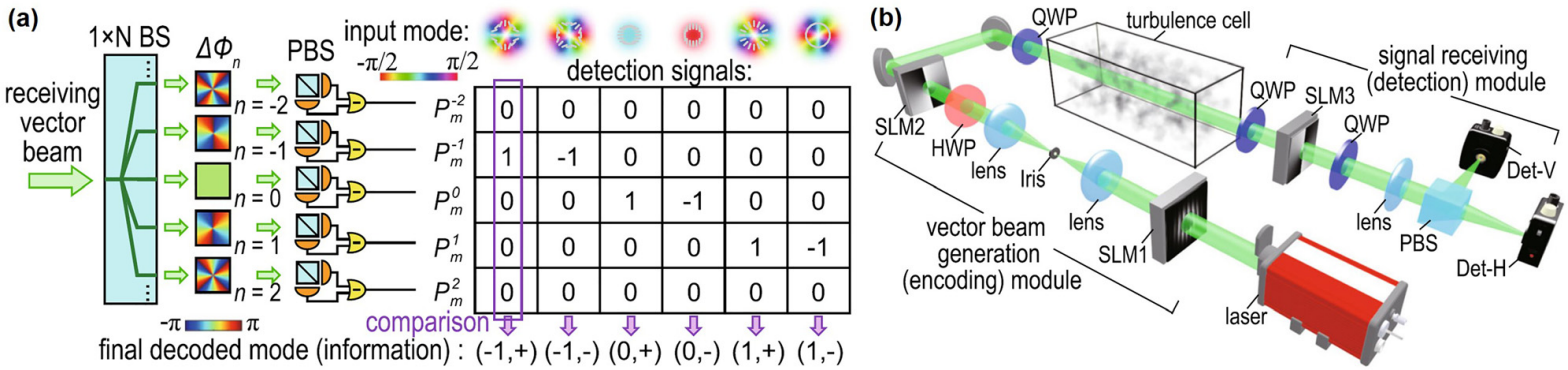
High-dimensional free-space optical communications using turbulence-resilient vector beams. (a) The principle of signal detection and (b) schematics of the experiment of high-dimensional spatial polarization differential phase shift keying (SPDPSK) [135]. Reprint permission obtained from [135].

A new high-dimensional communication protocol, namely, spatial polarization differential phase shift keying (SPDPSK) was demonstrated in Figure 26 [135]. The encoding and decoding of high-dimensional information was based on orthogonal spatial polarization states of a family of vector vortex beams. In the experiment, by using a carefully designed detection scheme as shown in Figure 26a, the spatial polarization profile of vector vortex beams could be resilient against atmospheric turbulence. At the receiver, the incoming optical signal was split into *N* copies. Each of them passed through a *n*th order polarization-dependent decoding phase mask before the differential power between H and V polarization was measured. All *N* decoded signals were then compared to determine the final detected information level (vector beam modes). The table on the right showed the detected signals of five decoding channels for six input modes (shown on the top with the color representing the polarization orientation), and the final decoded information level at the bottom. In this way, the SPDPSK protocol could transmit high-dimensional information reliably through a moderately strong turbulence cell with a scintillation index of up to 1.54 in the absence of any beam compensation mechanism. A proof-of-principle high-dimensional communication system was demonstrated in the experiment by transmitting 34 information levels (5.09 bits of information) per pulse through a free-space channel in the moderately strong turbulence regime with small information loss [135].

### 9.3 Intelligent communications using OAM and beyond

Machine learning (ML) is an interdisciplinary science combined with the mathematics, computer, and biology science, which have been employed in computer vision, natural language processing, data mining, and optical communications. To get intelligent optical communication systems, ML algorithms have been demonstrated in optical performance monitoring (OPM), nonlinear impairments compensation, modulation format identification and so on [136], [137], [138].

Recently, to achieve real artificial intelligence and high accuracy of object recognition, deep learning becomes a rapidly expanding research topic. As a core member in the deep learning models, convolutional neural network (CNN) has made great breakthroughs in the image recognition. There are four key characters making CNN distinct from the conventional neural networks: local connections, shared weights, pooling, and multi-layer structure. Benefitting from the above features, CNN has merits of directly recognizing raw images and discovering intrinsic features of input images without the careful feature extraction engineering. Very recently, the CNN with improved structure has been widely introduced into free-space optical communications exploiting spatial modes.

**Figure 27:**
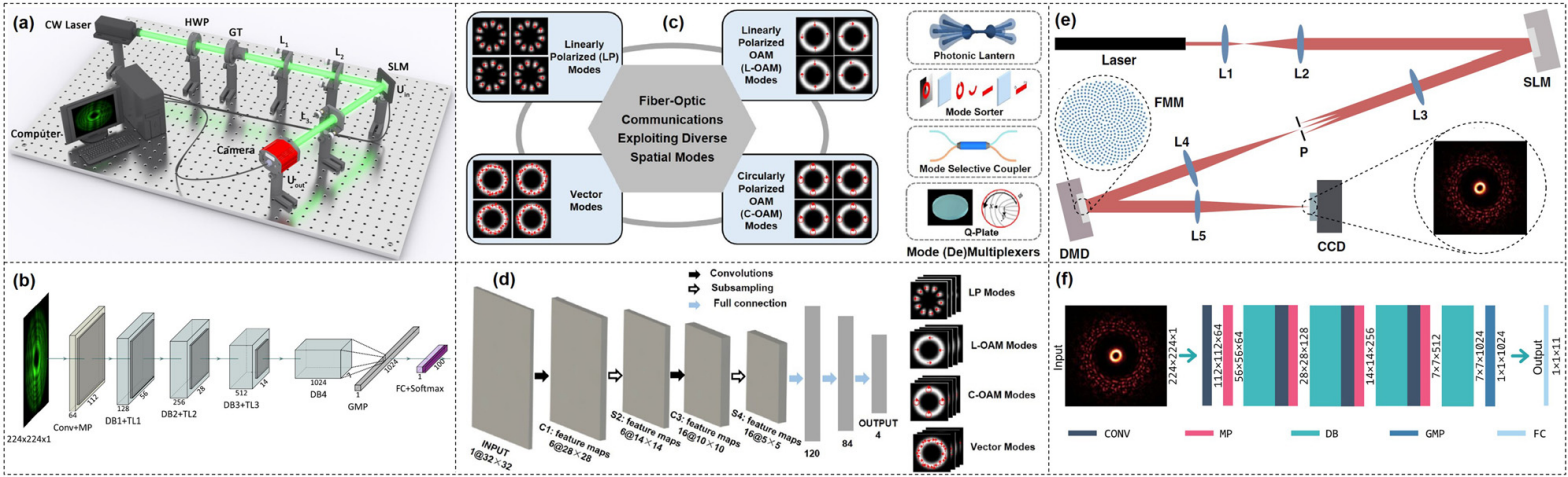
Intelligent spatial modes recognition in optical communications. (a) Experimental setup of OAM-recognition neuron network (ORNN) with deep learning. (b) Sketch map of the ORNN architecture to recognize OAM beams. Conv: convolution layer; MP: max pooling layer; DB: dense block; TL: transition layer; GMP: global max pooling layer; FC: fully connected layer [139]. (c) Illustration of fiber-optic communications exploiting diverse spatial mode bases and mode-base-dependent mode (de)multiplexer. (d) Schematic diagram of the specific structure of the convolutional neural network (CNN) used to recognize four kinds of mode bases (LP modes, L-OAM modes, C-OAM modes, vector modes). LP: linearly polarized; L-OAM: linearly polarized OAM; C-OAM: circularly polarized OAM [140]. (e) Alignment-free fractal multipoint interferometer. Laser: He–Ne laser with 633-nm wavelength; L1: 50-mm lens; L2: 500-mm lens; SLM: phase-only spatial light modulator; L3: 300-mm lens; P: pinhole; L4: 300-mm lens; DMD: digital micromirror device; L5: 250-mm lens; CCD: charge-coupled device.(f) Schematic diagram of DenseNet-121 [141]. Reprint permission obtained from [139–141].

As shown in Figure 27a, a deep learning method to precisely recognize OAM beams with fractional topological charges was proposed. The minimum interval recognized between adjacent modes reached to 0.01. A super high-resolution OAM multiplexing system transmitting Einstein portrait was implemented to evaluate the efficiency in the optical communication process. Figure 27b displayed the architecture of OAM-recognition neuron network (ORNN). Despite OAM beam recognition in free space, spatial mode bases recognition based on the CNN structure in fiber-optic communications are also desired. A CNN architecture, namely LeNet-5, was proposed in Figure 27c, which was applied to four mode bases recognition in the ring-core fiber (RCF) [140]. The intensity images of four familiar bases representing modes with azimuthal index *l* = 5, namely the LP_5,1_ mode group, the linearly and circularly polarized OAM_±5,1_ mode group, and the vector EH_4,1_ or HE_6,1_ mode group, were trained and tested using the proposed technique. Figure 27d displayed a schematic diagram of the specific structure of the CNN used to recognize different mode bases. The experimental results showed that CNN could effectively classify all these mode bases with an overall recognition rate of close to 100%. Moreover, a photodetector (PD) array for compact and cost-effective intelligent mode bases recognition was proposed. A 1 × 5 PD array could obtain a recognition rate of close to 100%, and even a 1 × 2 PD array with only two PDs could achieve a high recognition rate of close to 93.3%. Though OAM mode recognition has been studied widely, it is still a challenge to recognize OAM mode with high accuracy for misalignment of lateral displacement, beam waist size, and initial phase. Figure 27e proposed a deep learning method to exquisitely recognize OAM beams under misalignment by using an alignment-free fractal multipoint interferometer [141]. The wavefronts of OAM beams were sampled by a well-designed fractal multipoint mask (FMM). Thus, wealthy diffraction intensity patterns could be recorded for different OAM beams. Meanwhile, the diffraction patterns were stable against reference misalignment because of the inherent periodic structure of the FMM. The architecture of the DenseNet-121 was shown in Figure 27h.

**Figure 28:**
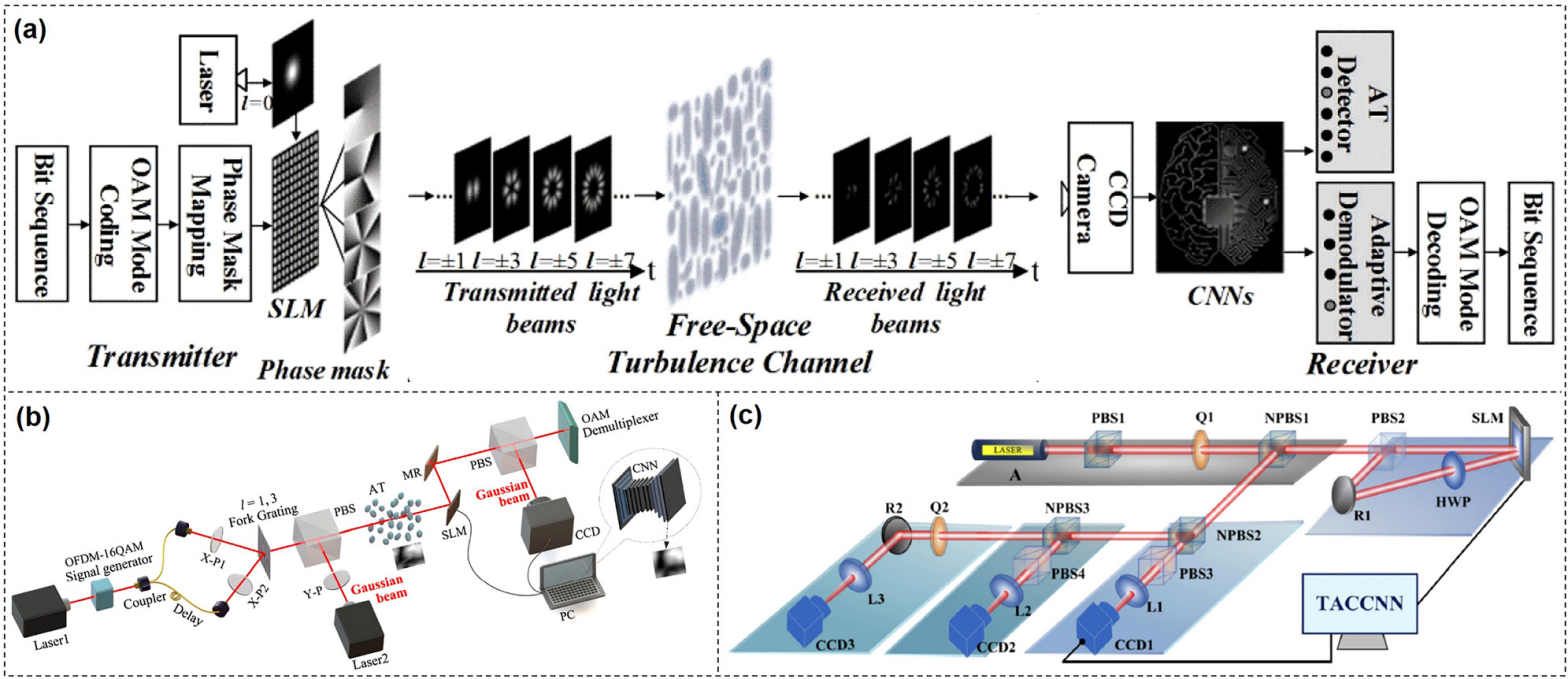
Intelligent spatial mode optical communications. (a) Joint atmospheric turbulence detection and adaptive demodulation technique based on CNN [142]. (b) The system diagram of OAM multiplexing communication link with atmosphere turbulence compensation assisted by the deep learning method [143]. (c) Experimental configuration using turbulence aberration correction convolutional neural network (TACCNN) for vector beam communications [144]. Reprint permission obtained from [142–144].

Besides OAM beams recognition, intelligent OAM communication has attracted extensive attentions. Figure 28a proposed a joint atmospheric turbulence detection and adaptive demodulation technique based on CNN [142]. Compared to previous approaches using the self-organizing mapping (SOM), deep neural network (DNN) and other CNNs, the proposed CNN achieved the higher atmospheric turbulence detecting accuracy (ATDA) and adaptive demodulating accuracy (ADA) due to the advanced multi-layer representation learning without feature extractors which was designed carefully by numerous experts. The ATDA and ADA of the 4-OAM, 8-OAM, 16-OAM free-space optical communication systems over computer-simulated 1000-m turbulent channels with 4, 6, 10 kinds of classic atmospheric turbulence were investigated, respectively. For turbulence compensation assisted by CNN, non-conjugate modes were uninvolved in the training, which decreased the correction efficiency as every superposition mode needed to be trained separately. Figure 28b proposed a CNN model, which could automatically learn the mapping relationship of the intensity distributions of input and the turbulent phase [143]. After trained with loads of studying samples, the CNN model possessed a good generalization ability in quickly and accurately predicting equivalent turbulent phase screen, including the untrained turbulent phase screens. Figure 28c displayed a deep learning based adaptive optics system to compensate the turbulence aberrations of the vector vortex mode in terms of phase distribution and mode purity [144]. A turbulence aberration correction convolutional neural network (TACCNN) model, which could learn the mapping relationship of intensity profile of the distorted vector vortex modes and the turbulence phase generated by first 20 Zernike modes, was well designed.

## 10 Conclusion and discussion

**Figure 29:**
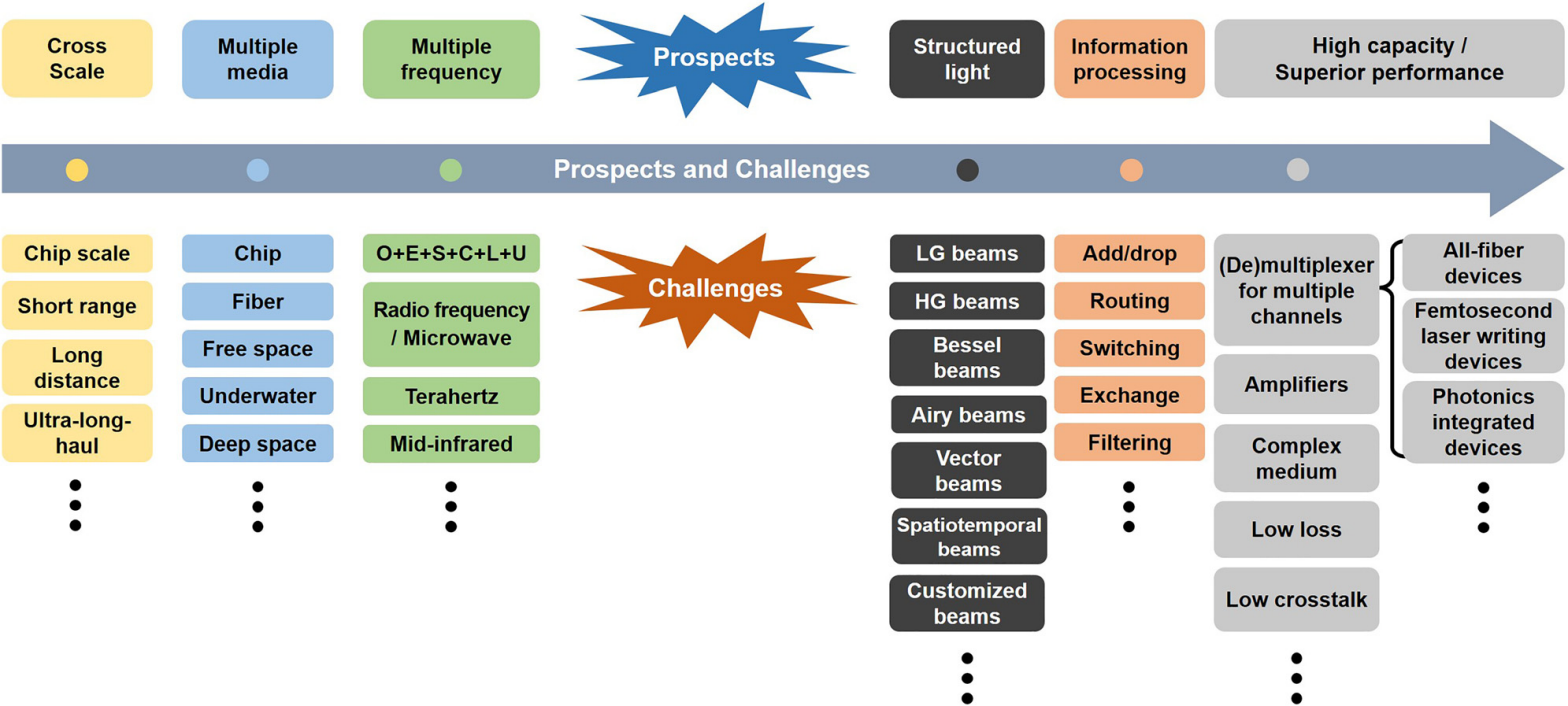
Prospects and challenges of optical communications using OAM and beyond.

In summary, we focus on optical communications using OAM and beyond in free space in this review article. The fundamentals of OAM, basic concept of optical communications using OAM, OAM modulation communications including OAM modulation based on SLM, high-speed OAM modulation and spatial array modulation, OAM multiplexing communications including spectrally-efficient, high-capacity and real-world long-distance free-space links, OAM multicasting including power-equalized, adaptive power controllable and *N*-dimensional 1-to-1100 multicasting operations, OAM communications in turbulence including compensation based on adaptive optics, digital signal processing and auto-alignment system, structured light communications beyond OAM including communications using Bessel beams, Airy beams and vector beams, diverse and robust OAM communications including OAM communications in multiple scenes, turbulence-resilient structured light communications and intelligent communications using OAM and beyond are comprehensively introduced and discussed. While OAM-carrying light and extended more general structured light have shown great potential and made important progress in free-space optical communications, there are still lots of challenges for future optical communications using OAM and beyond as shown in Figure 29.

**Figure 30:**
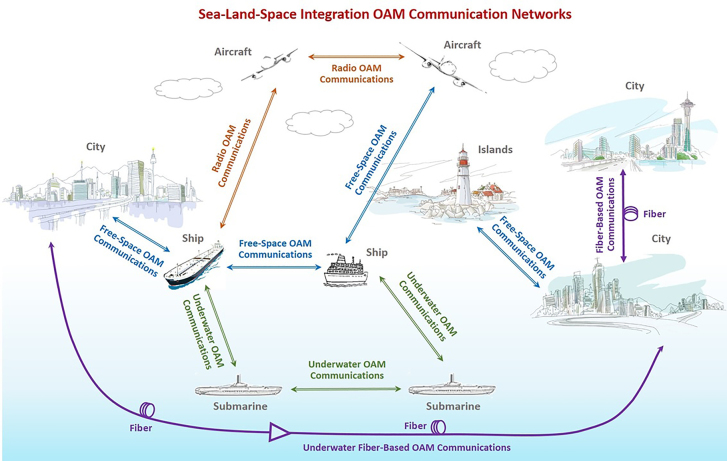
A vision of future OAM communication networks in multiple scenes.

OAM links cross different scales, e.g., chip scale, short range, long distance, and even ultra-long-haul transmission, are desirable in future diverse optical interconnects and communications. Remarkably, the hybrid integration of multiple scenes or media, e.g., chip, fiber, free space, underwater, and even deep space, are also of great importance and challenge [79, 131, 133, 145], [146], [147], [148], [149], [150], [151], [152]. Although OAM communications and beyond in a single scene have made remarkable progress in recent years, it is highly desirable to make different scenes seamlessly connected, for example, free space to underwater, free space to fiber, fiber to chip and so on, which is of great significance to the development of information globalization and integration. Besides, satellite-to-ground and satellite-to-satellite links may also employ OAM and beyond for communications. Figure 30 illustrates a vision of future sea-land-space integration OAM communication networks in multiple scenes.

At present, OAM optical communications are most for visible light or C-band (1530–1565 nm) infrared light. With future improvement, extending OAM optical communications towards the full O + E + S + C + L + U communication band larger than 400 nm (1260–1675 nm), i.e., O-band (1260–1360 nm), E-band (1360–1460 nm), S-band (1460–1530 nm), C-band (1530–1565 nm), L-band (1565–1625 nm), U-band (1625–1675 nm), will be the trend and quite challengeable. Moreover, OAM is a natural property of all electromagnetic waves beyond photons or light waves. Hence, the unique properties of OAM could be also applied to other electromagnetic spectrum ranges [153], [154], [155], including radio frequency/microwave, terahertz and mid-infrared, and their communications applications, e.g., radio OAM communications [156, 157] and sub-terahertz OAM communications [158].

Beyond OAM communications exploring the spatial phase structure, more general structured light beams with spatially variant amplitude/phase/polarization or spatiotemporal structure, e.g. LG beams, HG beams, Bessel beams, Airy beams, vector beams, spatiotemporal beams and arbitrarily customized beams, could also be employed in optical communications [73, 127, 159]. By accessing the full spatiotemporal structure physical dimensions of photons, we might have added opportunities to use different kinds of structured light in extensive applications.

In addition to OAM transmission links, OAM information processing is also the key technology for OAM-based optical communication networks, especially at the network nodes for flexible OAM management. Typical OAM information processing functions include OAM add/drop, OAM routing, OAM switching, OAM exchange, OAM filtering and so on [160], [161], [162], [163], [164]. Scalable, reconfigurable and intelligent OAM information processing applications are the trend and preferred to enable flexible data management and seamless connection between different OAM networks.

Towards future high-capacity communications, it is highly desired to further scale the orthogonal channels in OAM communications, i.e., number of OAM beams. One challenge would be the scalable and efficient OAM (de)multiplexing techniques and devices compatible to conventional single-mode fiber (SMF). The (de)multiplexing devices could be fabricated on different platforms, such as all-fiber devices, femtosecond laser writing devices, photonic integrated devices and so on [165], [166], [167], [168]. In addition to OAM (de)multiplexer, scalable and efficient (de)multiplexers for more general structured light beams are also of great importance. Moreover, high-performance amplifiers for OAM beams and beyond with high modal gain, low differential modal gain and broad bandwidth, such as erbium-doped fiber amplifiers and Raman amplifiers, are another great challenge [169, 170–173].

Towards future communications with superior performance, low-loss and low-crosstalk complex medium optical communications using OAM and beyond are still of great challenge due to diffraction, scattering, divergence, obstacle and turbulence. Polar codes, low-density parity-check codes, partially coherent light transmission, space/mode diversity receiver, digital signal processing, deep learning assisted adaptive optics for equalization, acquisition, pointing and tracking (APT) techniques could be potential solutions [149, 174, 175].

Despite the significant progress achieved in free-space optical communications using OAM and beyond [12, 22–24, 176–180], it is still in its infancy, with lots of challenges and key issues to be fully explored. In the future, there will be more opportunities in exploiting extensive advanced applications, from OAM beams to more general structured light, and from communications to manipulation, microscopy, metrology, nonlinear optics and quantum science [5–9, 19–21, 181–184].
